# Recent advances and future challenges in the bottom-up synthesis of azulene-embedded nanographenes

**DOI:** 10.3762/bjoc.21.99

**Published:** 2025-06-26

**Authors:** Bartłomiej Pigulski

**Affiliations:** 1 Faculty of Chemistry, University of Wrocław, 14 F. Joliot-Curie, 50-383 Wrocław, Poland

**Keywords:** azulene, nanographenes, non-alternant, non-benzenoid, polycyclic aromatic hydrocarbons

## Abstract

In recent years, significant progress has been made in the synthesis of various nanographenes incorporating non-benzenoid rings, expanding the scope of molecular design beyond all-hexagon polycyclic aromatic hydrocarbons (PAHs). Among these, π-conjugated scaffolds featuring embedded azulene units have gained considerable attention due to their unique optical and electronic properties. This review provides an overview of representative azulene-embedded nanographenes, with a particular focus on the synthetic strategies. Additionally, it explores selected aspects of aromaticity and spectroscopic properties.

## Introduction

The discovery of graphene and fullerenes has sparked a continuously growing interest in synthesis of new carbon-rich unsaturated molecules and materials [1]. Graphene is a revolutionary material with exceptional properties, driving advancements across various scientific, industrial, and technological fields like organic electronics [2], medicine [3], sensing [4] and energy storage [5]. Typically, bulk graphene is obtained using a top-down approach, where graphite is exfoliated using chemical or mechanical methods [6–7]. However, this method does not provide precise control over the structure of graphene and graphenoid materials, which is crucial for fine-tuning their properties. An alternative is the bottom-up approach where various nanographenes are synthesized form smaller building blocks via classical organic synthesis. This strategy enables precise control over the structure and topology, leading to the development of a vast array of benzenoid nanographenes, also known as polycyclic aromatic hydrocarbons (PAHs) [8–9]. PAHs can be considered molecular models of bulk graphene, offering invaluable insights into structure–property relationships in graphene and graphene-based materials.

Structural defects appear to be inevitable in real graphene and graphenoid structures. The presence of heteroatoms, dislocations and grain boundaries [10] has a significant impact on the properties of graphene [11]. From both fundamental and applied perspectives, a thorough understanding of these topological defects is of great importance. Consequently, the investigation of well-deﬁned defects in atomically precise and monodisperse nanographenes plays a unique role in engineering defects in graphene, helping to elucidate the structure–property relationships.

**Figure 1:**
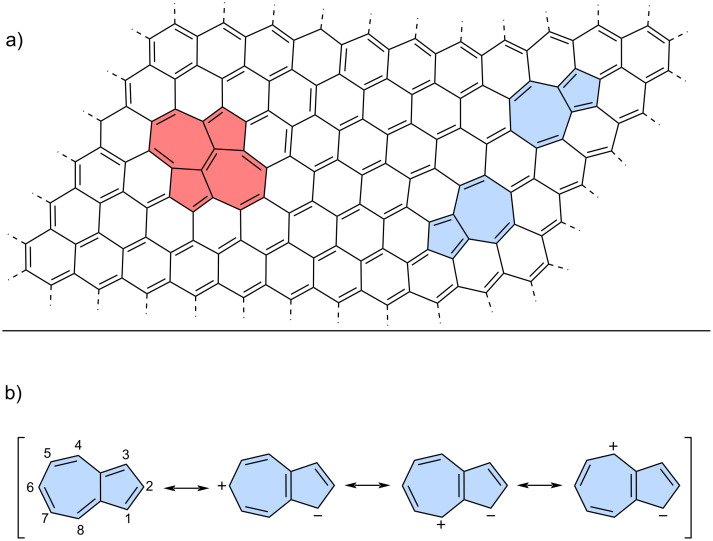
a) Stone–Wales (red) and azulene (blue) defects in graphene; b) azulene and its selected resonance forms.

Non-benzenoid rings are among the most important types of defects found in graphene [12–13] with Stone–Wales [14] and azulene [10] defects being the most representative examples (Figure 1a). Modelling and understanding these defects is a key motivation behind the growing interest in non-alternant, non-benzenoid PAHs [15]. In particular, the incorporation of azulene moieties into various PAHs is highly valuable, as such molecules provide deeper insights into structure–property relationships [16–17].

**Figure 2:**
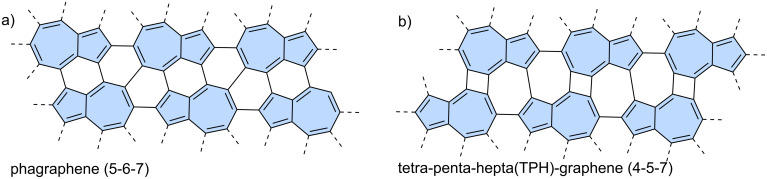
Examples of azulene-embedded 2D allotropic forms of carbon: a) phagraphene and b) TPH-graphene.

Azulene, an isomer of naphthalene, is the smallest non-alternant, non-benzenoid aromatic compound (Figure 1b). It consists of an electron-rich pentagon and an electron-deﬁcient heptagon, resulting in a significant dipole moment of 1.08 D [18]. Due to its unique non-alternant topology, azulene exhibits a smaller energy gap compared to that of isomeric naphthalene and unusual emission from the S_2_ state (*anti*-Kasha’s emission), as a consequence of its non-mirror related highest occupied molecular orbital (HOMO) and lowest unoccupied molecular orbital (LUMO) [19]. This distinctive behaviour gives rise to intriguing optoelectronic properties, making azulene an attractive candidate for practical applications. For example, graphene nanoribbons with azulene defects are promising materials for nonlinear optics (NLO) [20]. Furthermore, azulene subunits are present in many hypothetical allotropic two-dimensional carbon allotropes. In recent years many 2D graphenoid allotropic forms of carbon were theoretically predicted like a family based on the azulenoid kekulene [21], phagraphene (Figure 2a) [22], TPH-graphene (Figure 2b) [23], PHH-graphene [24] and ψ-graphene [25]. Notably, fragments phagraphene and TPH-graphene have already been synthesized via on-surface chemistry and characterized using low-temperature scanning probe microscopy with CO-functionalized tips [23]. These non-alternant carbon allotropes represent promising candidates for novel carbon-based materials with exotic properties.

Given the points discussed above, it is evident that exploring of synthetic pathways for azulene-embedded nanographenes is a highly relevant and important topic in contemporary synthetic organic chemistry. These well-defined molecules are not only valuable as fundamental models of defective graphene but also hold significant potential in organic electronics [26–27] despite the considerable challenges in their synthesis [28–29].

One important factor should be considered regarding azulene-embedded nanographenes. In the literature terms such as “azulene-embedded nanographenes” or “azulene-embedded PAHs” generally refer to any conjugated carbon scaffold composed of sp^2^ carbons with adjacent pentagonal and heptagonal rings. However, in many cases, the distinctive electronic structure of azulene is absent due to the dominance of surrounding benzenoid rings or the presence of biradical character. As a result, these PAHs despite, possessing formal azulene may exhibit properties typical of benzenoid molecules rather than the characteristic azulene features such as red-shifted absorption, a small HOMO–LUMO gap, aromaticity of azulene subunit and *anti*-Kasha’s emission from higher excited states. In such cases, the azulene unit merely acts as a linker within a more complex benzenoid framework.

This review covers all types of azulene-embedded molecular scaffolds, regardless of whether they contain a "formal" or "true" azulene subunits. However, one of the key objectives here is to highlight the differences between these structural types and provide a clear distinction between benzenoid structures with azulene-like linkers and molecules that can be considered “true” aromatic π-extended azulenes. This is why, in many cases, the aromaticity of the azulene moiety is discussed, particularly through the analysis of the most used variations of NICS (nucleus-independent chemical shifts) parameters [30]. Additionally, whenever possible, information on the wavelength of the lowest-energy optical transition is included, as it serves as an important indicator of the electronic structure.

This review provides an up-to-date summary of known synthetic strategies for azulene-embedded polycyclic aromatic hydrocarbons (PAHs) as models of defective graphene, offering guidelines for designing new carbon scaffolds of this type. Given the rapid progress in this field, with nearly half of the cited works published since 2020, this review focuses primarily on purely hydrocarbon structures, with less emphasis on heteroatom-containing molecules. Typically, only the final synthetic steps leading to the fused structures are discussed. However, in cases where it provides valuable context, key reactions leading to direct precursors are also described.

## Review

### Early approaches to azulene-embedded nanographenes

**Scheme 1:**
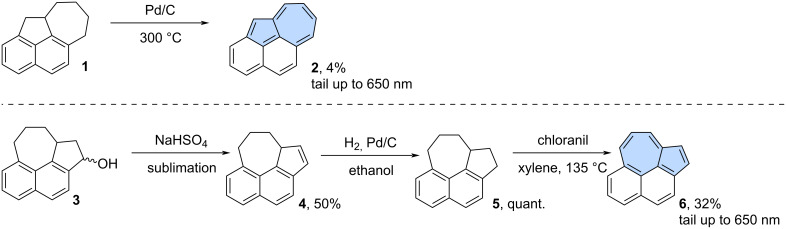
Synthesis of non-alternant isomers of pyrene (**2** and **6**) using dehydrogenation.

The following section provides a short historical overview of synthetic approaches leading to smaller purely hydrocarbon-based azulene-embedded nanographenes. The synthesis of smaller non-alternant PAHs containing azulene moiety dates to the 1950s. The most common strategy involved synthesizing a partially saturated scaffold, which was then dehydrogenated in the final step. One of the earliest examples of the synthesis of π-extended azulene was the non-benzenoid isomer of pyrene published by Ward and co-workers (Scheme 1) [31]. Cyclohept[*bc*]acenaphthylene (**2**) was obtained from a partially saturated precursor **1** via dehydrogenation using palladium on carbon. However, the reaction carried out at 300 °C gave **2** as a red solid in only 4% yield. A similar strategy was used by Osborn for the synthesis of isomeric compound **6** (Scheme 1) [32]. In this case, compound **3** was dehydrogenated giving compound **4** which was then reduced to the direct precursor **5**. Subsequent oxidation using chloranil yielded cyclohepta[*klm*]benz[*e*]indene (**6**) as black plates in a 32% yield. The azulene-containing isomers of pyrene exhibit azulene-like absorption tailing up to around 650 nm. Interestingly, despite their initial synthesis in the 1950s, no further attempts have been made to synthesize compounds **2** and **6** using more modern methods.

**Scheme 2:**
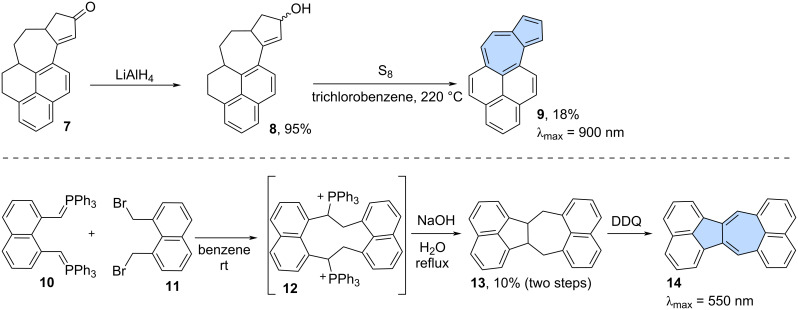
Synthesis of non-alternant isomer **9** of benzo[*a*]pyrene and **14** of benzo[*a*]perylene using dehydrogenation.

Dehydrogenation played a pivotal role as a key step also in the synthesis of larger π-scaffolds. For example, Murata and co-workers reported the synthesis of an azulene containing isomer of benzo[*a*]pyrene **9** (Scheme 2) [33]. Reduction of ketone **7** using LiAlH_4_ resulted in alcohol **8** which was subsequently dehydrogenated using sulfur in trichlorobenzene at 220 °C to yield the azulene-containing isomer of benzo[*a*]pyrene **9** in 18% isolated yield. Bestmann and Ruppert reported the synthesis of a dinaphthoazulene **14**, a non-alternant isomer of benzo[*a*]perylene (Scheme 2) [34]. In their method, bisylide **10** was reacted with dibromide **11** to form cyclic bisphosphonium salt **12,** which was then subjected to alkaline hydrolysis. The direct precursor **13** was isolated in 10% yield after two steps and, finally, oxidized to PAH **14** using DDQ (2,3-dichloro-5,6-dicyano-1,4-benzoquinone).

**Scheme 3:**
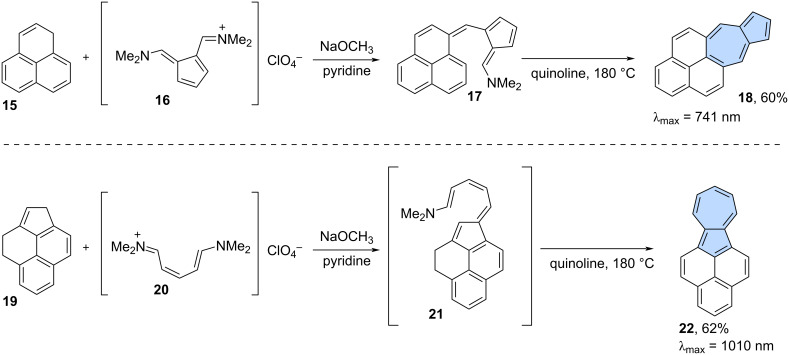
Synthesis of azulene-embedded isomers of benzo[*a*]pyrene (**18** and **22**) inspired by Ziegler–Hafner azulene synthesis.

The second approach was inspired by well-known Ziegler–Hafner azulene synthesis [35]. The key step in this method involves the synthesis of the intermediate pentafulvene, which is subsequently cyclized to yield the target azulene. An example of this strategy is the synthesis of the azulene-embedded isomer of benzo[*a*]pyrene which was reported by Jutz and Kirchlechner in 1966 (Scheme 3) [36]. Condensation between phenalene **15** and pentafulvene **16** gave pentafulvene **17**. Pentafulvene **17** was finally subjected to Ziegler–Hafner reaction in quinoline at 180 °C, resulting in the π-extended azulene **18** in 60% yield. A similar synthetic strategy was employed by Hara and co-workers in 1975 (Scheme 3) [37]. Compound **19** reacted with cyanine **20** to give pentafulvene **21**. Compound **21** was later cyclized in quinoline at 180 °C giving non-alternant isomer of benzo[*a*]pyrene **22** in 62% isolated yield. PAH **22** has remarkable re-shifted optical absorption with λ_max_ = 1010 nm, which is a strong indicator of its dominant non-alternant character.

Traditional methods for synthesizing azulene-embedded PAHs often require harsh conditions, making them challenging to apply to larger π-scaffolds. Furthermore, these methods frequently suffer from low yields and are not easily adapted to more modular approaches, limiting the variety of possible substitution patterns. This is why more modern approaches continue to be developed.

### Modern approaches to azulene-embedded nanographenes

**Figure 3:**
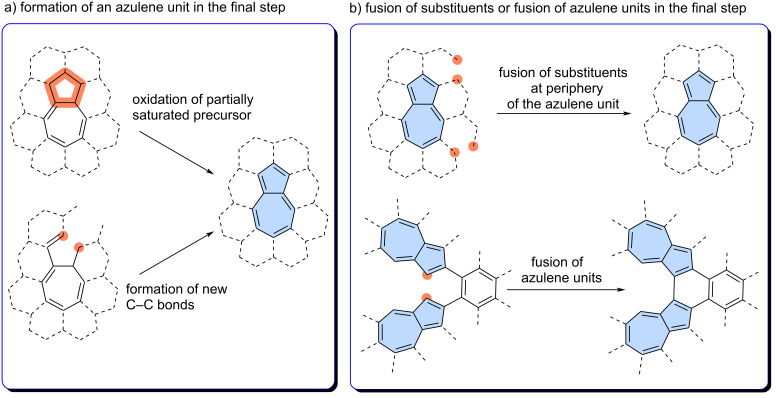
General strategies leading to azulene-embedded nanographenes: a) construction of azulene moiety in the final step: b) fusion of substituents at the periphery of fusion of azulene units.

Modern synthetic approaches have greatly benefited from the discovery of palladium-catalysed cross-coupling reactions, such as the Suzuki sp^2^–sp^2^ coupling or Sonogashira sp^2^–sp coupling. These reactions enable the modular construction of complex precursors, which can then be transformed into azulene-embedded PAHs in the final step. Two main synthetic strategies are commonly employed: 1) The construction of the azulene moiety in the final step by creation of new C–C bond(s) or oxidation of a partially saturated precursor (Figure 3a); 2) The use of precursors that already contain the azulene moiety or moieties, which are then annulated into fully fused PAHs in the final step (Figure 3b). Obviously, the synthesis of more complex molecules may require elements of both strategies.

#### Construction of the azulene moiety in the final step

**Oxidation of partially saturated precursors:** With modern cross-coupling reactions providing access to larger precursors, a synthetic strategy involving the dehydrogenation of partially unsaturated precursors in the final stage can be applied to larger molecules as well. In this approach, the final PAHs with embedded formal azulene moieties are formed from substrates that already possess adjacent heptagons and pentagons but are partially saturated [38].

**Scheme 4:**
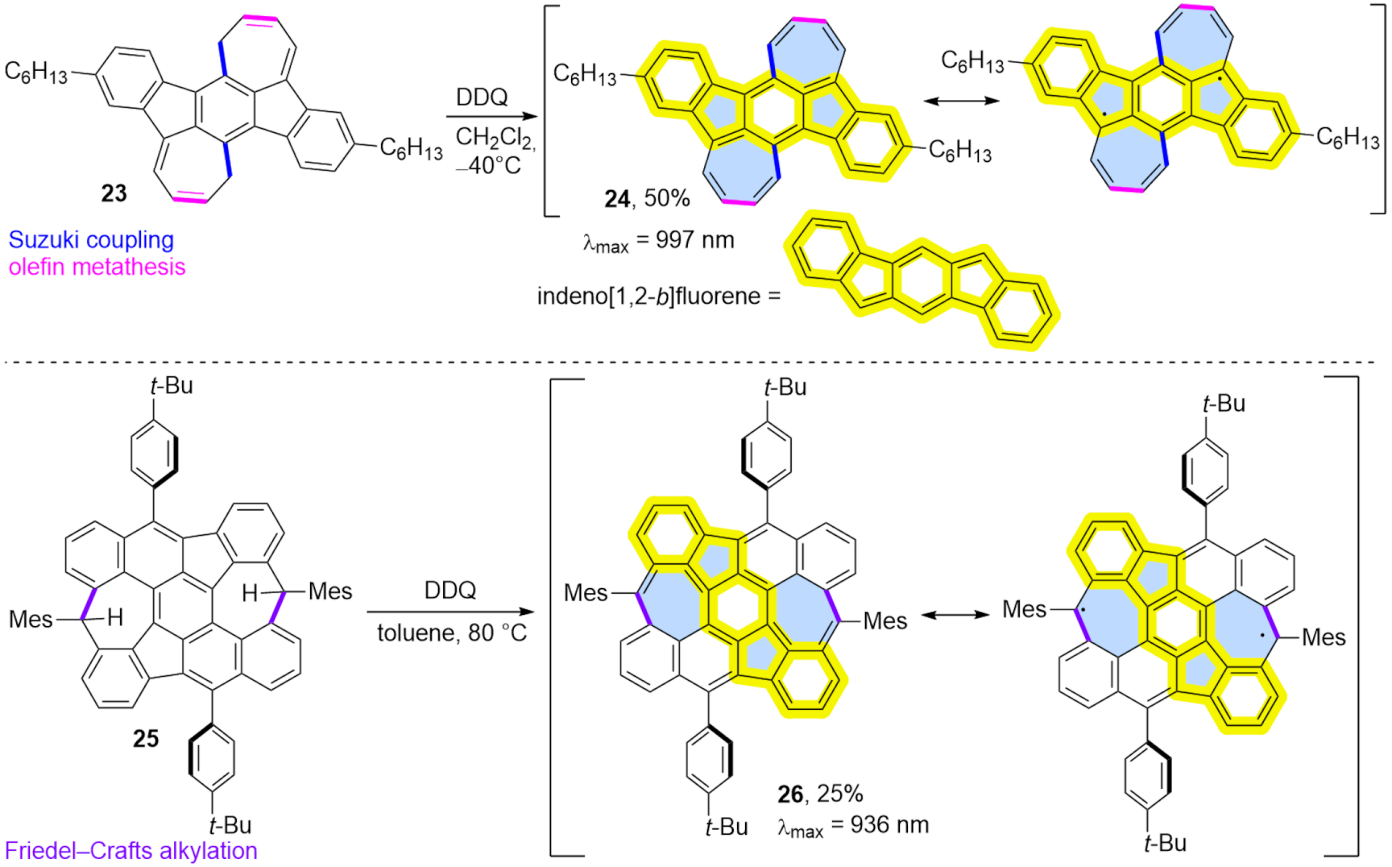
Synthesis of biradical PAHs possessing significant biradical character using oxidation of partially unsaturated precursors.

Ie, Aso and co-workers reported the oxidation of partially saturated precursor **23** using DDQ, which led to the isolation of PAH **24** in 50% yield which contains two formal azulene units (Scheme 4) [39]. However, compound **24** was found to possess a biradical structure (biradical character index, *y*_0_ = 0.49) with antiaromatic character of the pentagon, in contrast to pristine azulene. This results in a significantly red-shifted optical absorption at 997 nm. Therefore, compound **24** should be considered a formally antiaromatic extended indeno[1,2-*b*]fluorene, rather than a ‘true’ extended azulene.

Similarly, Müllen and co-workers reported the synthesis of non-benzenoid open-shell nanographene **26** from partially saturated precursor **25** in 25% yield after oxidation using DDQ (Scheme 4) [40]. Extensive characterization of the resulting nanographene in solution revealed a low optical gap, and an open-shell singlet ground state with a low singlet−triplet gap. Nanographene **26**, which also contains the indeno[1,2-*b*]fluorene structural motif, displays an extremely narrow energy gap of 0.27 eV and exhibits a pronounced open-shell biradical character, with biradical character index close to 1 (*y*_0_ = 0.92). Very recently, a similar synthetic strategy was used by Jiang and co-workers for the synthesis of very stable non-alternant nanographene with a triplet ground state [41].

**Scheme 5:**
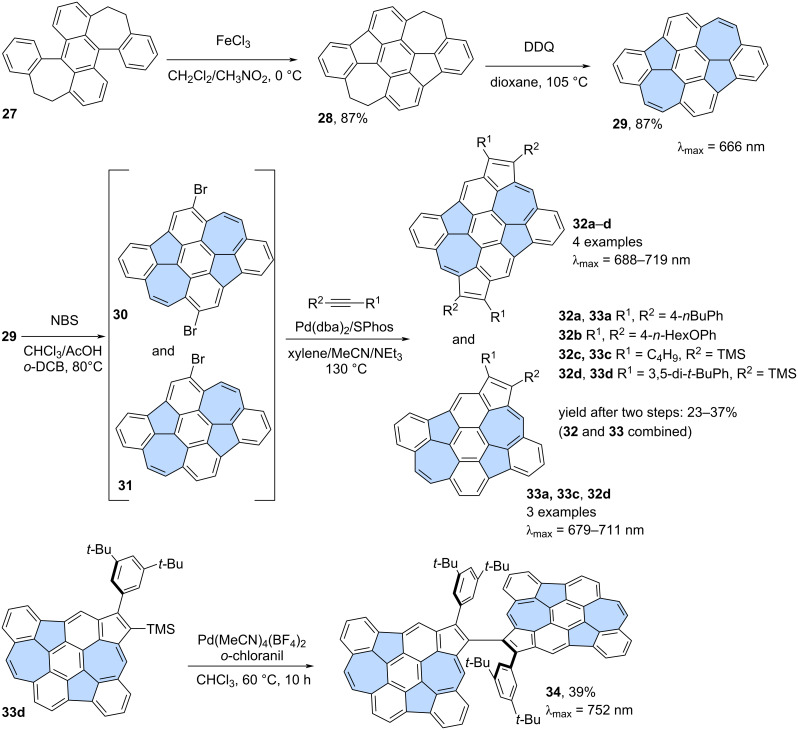
Synthesis of dicyclohepta[*ijkl*,*uvwx*]rubicene (**29**) and its further modifications.

Zhang and co-workers reported the synthesis of diazulenorubicene **29**, a non-benzenoid isomer of *peri*-tetracene (Scheme 5) [42]. The stepwise oxidation of compound **27** [43] which first yields the partially saturated product of the Scholl reaction (**28**) when FeCl_3_ in CH_2_Cl_2_/MeNO_2_ was used as an oxidant. Further oxidation was possible using DDQ in 1,4-dioxane and finally fully unsaturated PAH **29** was isolated in 87% yield. Compound **29** is a substructure of PAH **26** but does not exhibit biradical character. Instead, heptagons and pentagons are primarily non-aromatic, with a localized double C=C bond in the seven-membered ring, giving compound **29** predominantly benzenoid properties. Interestingly, PAH **29** undergoes single or double bromination with NBS in a mixture of CHCl_3_, AcOH and *o*-DCB [44]. The resulting mixture of brominated PAHs **30** and **31** was then subjected to single or double [3 + 2] annulation with various alkynes, leading to the extended structures **32** and **33**. Notably, compound **33d** can undergo Pd-catalysed dimerization, resulting in the formation of a chiral non-benzenoid nanographene **34** [45]. Single crystals of **34** surprisingly exhibit SHG-CD (second harmonic generation–circular dichroism) properties due to the unusual self-sorting of *R* and *S* enantiomers in the crystalline state.

**Scholl-type oxidation:** The Scholl oxidation is a highly useful tool for constructing various benzenoid polycyclic aromatic hydrocarbons (PAHs) [9]. So it is not a surprise that such fusion reactions were used in construction of azulene embedded in various nanographenes. However, when applied to complex and sterically crowded precursors, the reaction often involves a degree of unpredictability. Additional rearrangements and substitutions might occur making the exact outcome of the reaction difficult to predict.

**Scheme 6:**
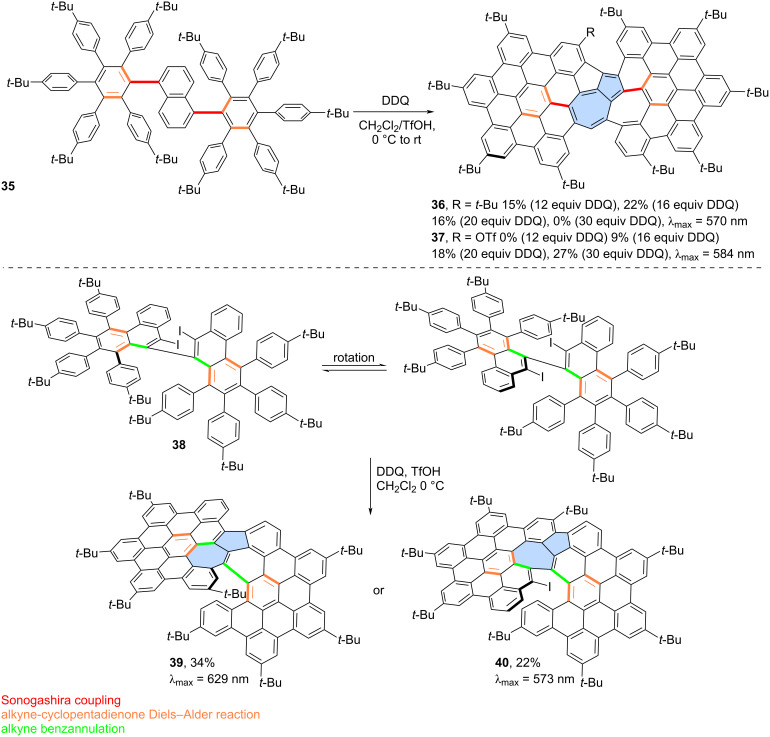
Synthesis of warped PAHs with one embedded azulene subunit using Scholl-type oxidation.

For example, Chi and co-workers unexpectedly obtained azulene-embedded nanographene **36** and its triflyloxylated derivative **37** from precursor **35** during the an attempted synthesis of a naphthalene-bridged double [6]helicene (Scheme 6) [46]. Depending on the amount of DDQ used for oxidation, the yield of **36** reached up to 22%, while **37** was obtained in up to 27% yield. The proposed mechanism for the formation of **36** and **37** involves an arenium ion-mediated 1,2-phenyl shift followed by a naphthalene-to-azulene rearrangement. The alternative radical cation mechanism has a higher energy barrier than the arenium cation-mediated reaction. Notably, only one of the pentagon–heptagon pairs exhibits an azulene-like electronic structure and aromaticity, as confirmed by the analysis of calculated NICS values. Similarly, Liu and co-workers reported the synthesis of two related nanographenes from precursor **38** (Scheme 6) [47]. Oxidation using DDQ/TfOH yielded two PAHs **39** and **40** in 34% and 22% yield, respectively. The authors postulated here formation of azulene moiety through radical cation mechanism and 1,2-phenyl shift. However, in this case, analysis of NICS values indicated that the azulene moiety does not exhibit aromatic character, and the electronic properties of the final molecules are primarily determined by the surrounding benzenoid rings.

**Scheme 7:**
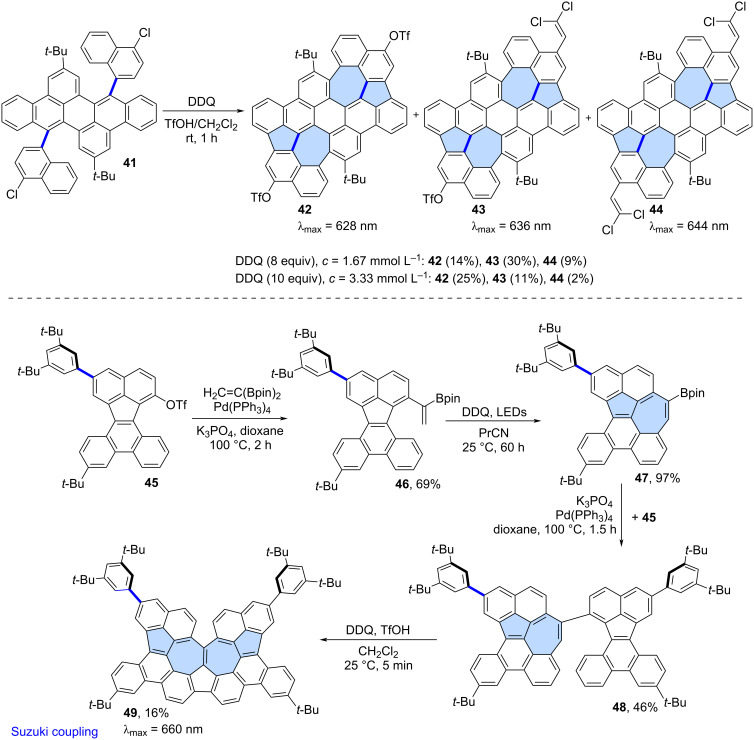
Synthesis of warped PAHs with two embedded azulene subunits using Scholl oxidation.

Mastalerz and co-workers reported the oxidation of precursor **41** using DDQ which led to a mixture of azulene-embedded PAHs **42**–**44** (Scheme 7) [48]. Contorted PAHs **42**–**44** containing two azulene subunits, were formed through a single-step cyclopentannulation and cycloheptannulation process. The cyclodehydrogenation reaction was accompanied by further regioselective functionalization at the periphery of the PAHs. Besides triflyloxylation (**42**, **43**), the introduction of one or two dichlorovinylene groups (**43**, **44**) was observed. As in previous cases, the exact ratio of the products depended on the amount of DDQ and concentration of the precursor. Analysis of NICS values of **42**–**44** revealed that the “formal azulene” units do not exhibit aromatic character, and the electronic properties of the molecules are primarily determined by the surrounding benzenoid rings.

Takasu and co-workers reported a more complex application of Scholl-like oxidation for the construction of an azulene-embedded nanographene **49**, featuring embedded contiguous azulene units and a narrowed cove-type edge (Scheme 7) [49]. Compound **46** was first subjected to intramolecular oxidation using light-promoted DDQ and as the result the first azulene subunit was introduced giving PAH **47** in an exceptionally high yield (97%). A Suzuki cross-coupling reaction between **47** and **45** gave compound **48** which was subjected to a final Scholl oxidation using DDQ. The target compound **49**, containing two azulene subunits, was obtained in a relatively low yield (16%). Analysis of NICS values for **49** revealed similar characteristics to most azulene-embedded PAHs obtained via Scholl oxidation – specifically, the azulene subunit does not exhibit aromaticity, and the surrounding benzenoid rings predominantly determine the electronic structure of **49**.

**Scheme 8:**
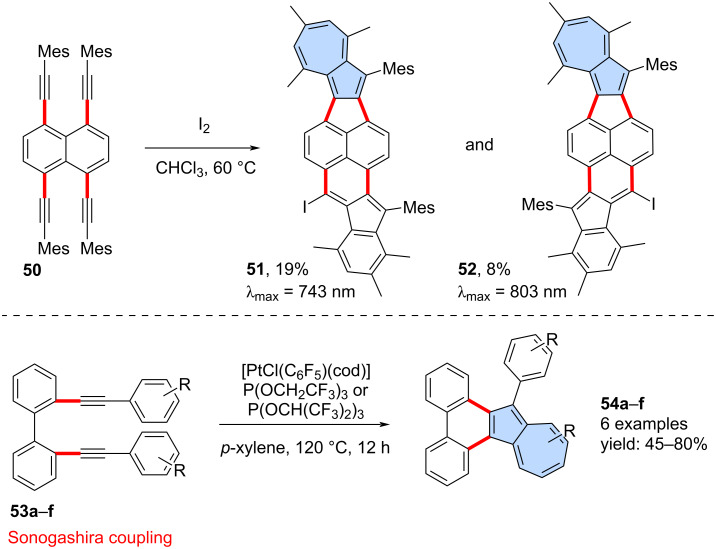
Synthesis of azulene-embedded PAHs using [3 + 2] annulation accompanied by ring expansion.

**[3 + 2] Annulation of alkynes accompanied by a phenyl ring expansion:** The dimerization of alkynes, followed by the expansion of a phenyl ring leading to the formation of an azulene moiety, was first reported over half a century ago. These reactions can be carried out using various catalytic systems, including sulfenyl chloride/AlCl_3_ [50], palladium catalysts [51] or gold catalysts [52]. With the appropriate choice of substrates, this approach can also be used for the synthesis of π-extended azulenes. For example, Tobe and co-workers conducted the intramolecular cyclization of 1,4,5,8-tetrakis(mesitylethynyl)naphthalene **50** using I_2_ in CH_2_Cl_2_ (Scheme 8) [53]. In the reaction resulted in the mixture of isomeric π-extended azulenes **51** and **52** in rather moderate yields of 8% and 8%, respectively. Both PAHs **51** and **52** contain also embedded non-alternant indenophenalene subunits. Similarly, Murakami and co-workers reported intramolecular dimerization of alkynes followed by a phenyl ring expansion for 2,2’-di(arylethynyl)biphenyls **53a**–**f** [54]. The platinum-catalysed reaction led to a series of azulenophenanthrens **54a**–**f** in yields ranging from 40% to 80%.

**Scheme 9:**
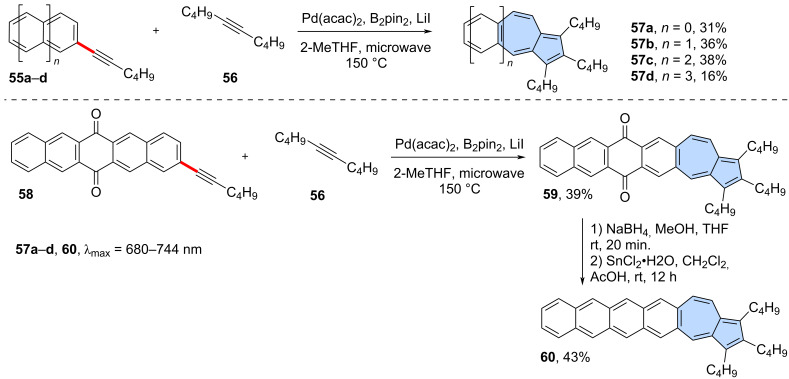
Synthesis of azulene-embedded isomers of linear acenes using [3 + 2] annulation accompanied by ring expansion.

A similar strategy was employed very recently in the synthesis of azulene-embedded isomers of linear acenes (Scheme 9) by Zhang and co-workers [55]. A palladium-catalysed variation of [3 + 2] annulation, accompanied by ring expansion [56], was used for the intermolecular reaction between acenes bearing alkyne substituents **55a**–**d** and di-*n*-butylacetylene (**56**). The reaction gave a series of azulene-embedded isomers of linear acenes from anthracene to pentacene (**57a**–**d**) in rather low yields (16–38%). The synthetic pathway leading to the hexacene isomer **60** was more complex due to the high reactivity of intermediate pentacenes. Instead, pentacene-6,13-dione **58** was subjected to the reaction with di-*n*-butylacetylene (**56**) giving azulene-embedded dione **59** in 39% yield. Finally, NaBH_4_ reduction followed by SnCl_2_/AcOH dehydration gave target non-alternant isomer of hexacene **60** in 43% yield. Interestingly, for the same number of rings, azulene-embedded acene isomers isomers exhibit greater stability than their fully benzenoid acene counterparts. Moreover, the azulene-like electronic structure is preserved, leading PAHs **57a**–**d** and **60** to display characteristic low-energy azulene absorption and *anti*-Kasha emission.

**Scheme 10:**
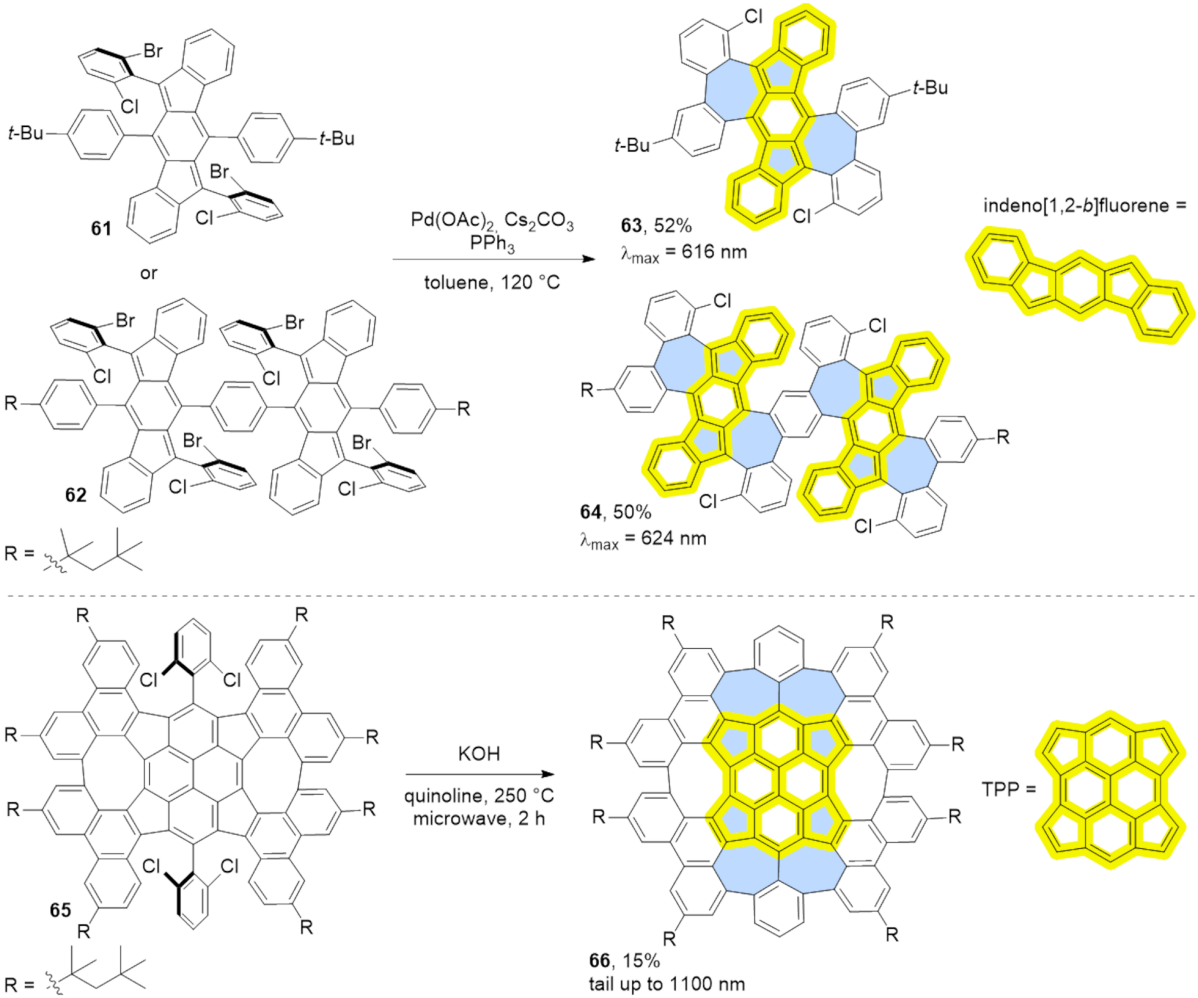
Synthesis of azulene-embedded PAHs using intramolecular C–H arylation.

**Intramolecular C–H arylation:** Various C–H arylation strategies have proven to be effective as the final step in the synthesis of azulene-embedded PAHs. This approach requires a halogen-functionalized precursor and typically employs a palladium catalyst. Dou and co-workers reported a last-stage intramolecular C–H arylation of substituted indenofluorenes **61** and **62** (Scheme 10) [57]. The palladium-catalysed reaction yielded fused products containing either two (**63**) or four azulene subunits (**64**). Analysis of NICS values revealed that the formally antiaromatic character indeno[1,2-*b*]fluorene units remain dominant in fused PAHs **63** and **64** resulting also in a biradical character. Considering these factors, PAHs **63** and **64** should be regarded as extended indenofluorenes that contain only “formal azulene” subunits rather than exhibiting true azulene-like electronic properties.

Zhang and co-workers reported the synthesis C_84_ molecular carbon **66**, which contains 10 non-benzenoid rings including four “formal azulene” units (Scheme 10) [58]. Interestingly, initial attempts to convert **65** into **66** using various procedures for the palladium-catalysed C–H activation were unsuccessful, even when conducted at elevated temperatures. However, treatment of **65** with KOH in refluxing quinoline successfully yielded the desired PAH **66**, albeit in a modest 15% yield. Despite the presence of four “formal azulene” units, the TPP (tetracyclopenta[*cd*,*fg*,*jk*,*mn*]pyrene) core of compound **66** exhibits antiaromatic properties. As a result, the pentagons within the structure remain antiaromatic, while the heptagons are non-aromatic, indicating the absence of an azulene-like electronic structure.

**Scheme 11:**
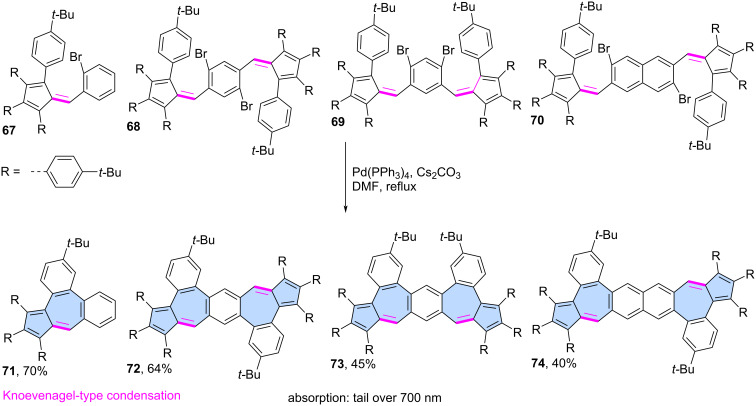
Synthesis of azulene-embedded isomers of acenes using intramolecular C–H arylation.

Liu and co-workers developed a modular approach to for synthesizing azulene-embedded isomers of linear acenes (Scheme 11) [59]. Precursors **67**–**70** were obtained from aldehydes and substituted cyclopentadienes using Knoevenagel-type condensation. Finally, intramolecular palladium-catalyzed C–H arylation afforded the fused azulene-embedded PAHs **71**–**74** in good yields (40–70%). All non-alternant isomers of linear acenes exhibit azulene-like lowest energy optical absorption, attributed to the azulene-like S₀→S₁ transition.

**Scheme 12:**
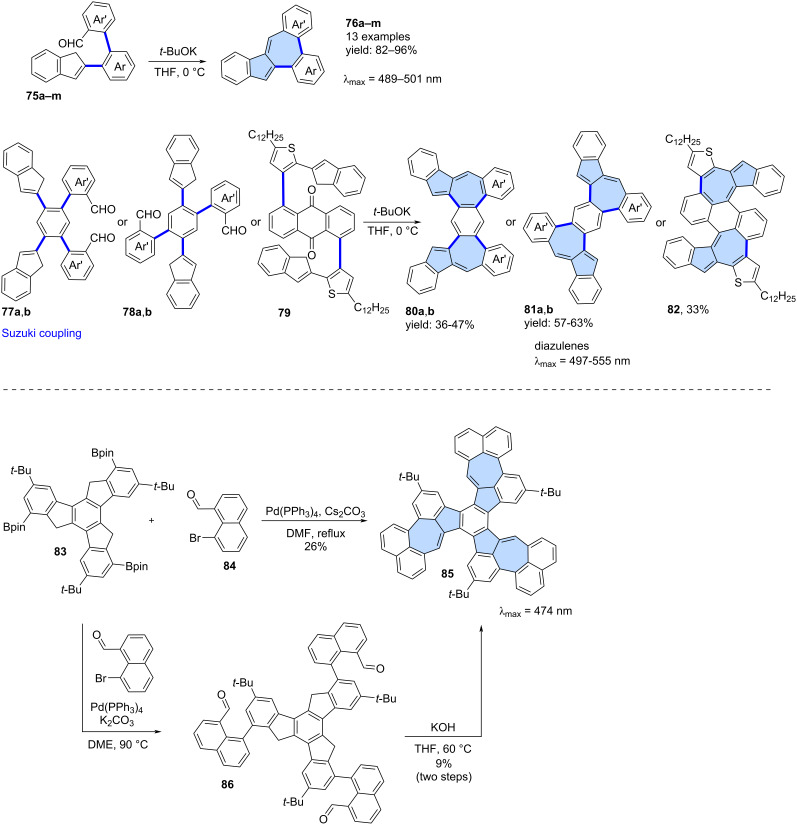
Synthesis of azulene-embedded PAHs using intramolecular condensations.

**Condensation reactions:** Various condensation reactions can also serve as valuable synthetic tools for constructing azulene subunits in the final step. For example, Ma and co-workers reported a modular synthetic strategy for the synthesis of diverse azulene-embedded PAHs via a tandem Suzuki coupling and base-promoted Knoevenagel-type condensation, achieving good yields and high structural versatility (Scheme 12) [60]. In this approach, precursors **75a**–**m** were first obtained using a Suzuki cross-coupling and subsequently subjected to the Knoevenagel-type condensation using *t-*BuOK. As a result, 13 PAHs **76a**–**m** containing an azulene subunit were synthesized in very good yields (82–96%). This strategy was later extended to larger molecules incorporating two azulene subunits. Precursors **77a**,**b**, **78a**,**b** and **79** underwent a similar intramolecular condensation, yielding PAHs with two azulene subunits (**80a**,**b**, **81a**, **81b** and **82**) in yields ranging from 33% to 63%. Analysis of NICS values for the resulting PAHs revealed that the azulene subunits did not exhibit typical azulene-like aromaticity. Notably, this represents one of the few modular approaches to azulene-embedded nanographenes that enables the synthesis of a larger family of molecules using a unified synthetic strategy.

The tandem Suzuki coupling/Knoevenagel condensation strategy leading to PAH **85** was independently reported by Liu [61] and Mastalerz (Scheme 12) [62]. The first group reported a cascade formal [3 + 4] annulation between triple boronic ester **83** and naphthalene **84** which combines a Suzuki cross-coupling and Knoevenagel-type condensation in a single synthetic step. This transformation was facilitated by Cs₂CO₃, which acted as a base for both the coupling and condensation reactions, ultimately yielding nanographene **85** in 26%. In contrast, Mastalerz and co-workers used a two-step strategy where the product of the Suzuki coupling **86** was first isolated. Compound **86** was then subjected to condensation with KOH in THF, affording **85** in an overall 9% yield over two steps. Additionally, the group reported an alternative synthetic route via trioxobenzotrisazulene, achieving a 25% total yield of **85** over three steps. It is worth noting that an alternative synthetic route via trioxobenzotrisazulene was also developed, achieving a 25% total yield of **85** over three steps [61–62].

**Scheme 13:**
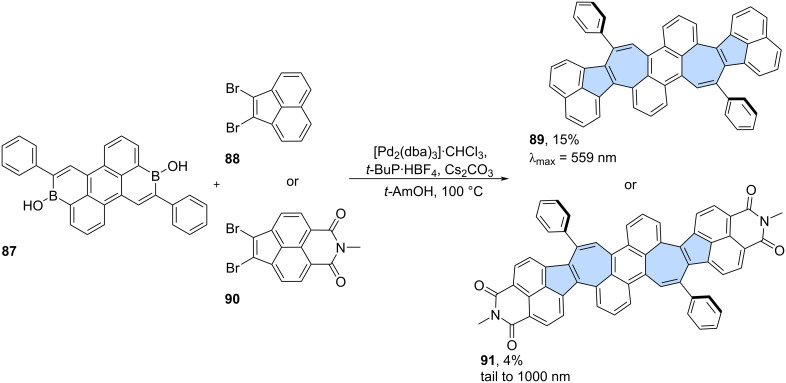
Synthesis of azulene-embedded PAH **89** using palladium-catalysed [5 + 2] annulation.

**Miscellaneous reactions:** Less conventional reactions can also serve as valuable synthetic tools for constructing "formal azulene" subunits in the final step. Würthner and co-workers utilized a palladium-catalysed [5 + 2] annulation reaction which was developed in the group [63]. This strategy has been demonstrated previously as an efficient approach for constructing sp^2^-hybridized heptagons. In their study, a two-fold palladium-catalyzed [5 + 2] annulation was performed using 3,9-diboraperylene [64] **87** and 1,2-dibromoacenaphthylene **88**, yielding the azulene-embedded PAH **89** with an isolated yield of 15% (Scheme 13) [65]. While the azulene subunits in **89** were shown to be antiaromatic in the neutral PAH, oxidation to the dication induced an aromaticity switch, resulting in the pentagon–heptagon pair adopting an aromatic character. The group later extended this strategy to scaffold **91** decorated with two imide substituents, which was isolated in 4% yield [66].

#### Annulation of substituted azulenes

**Scheme 14:**
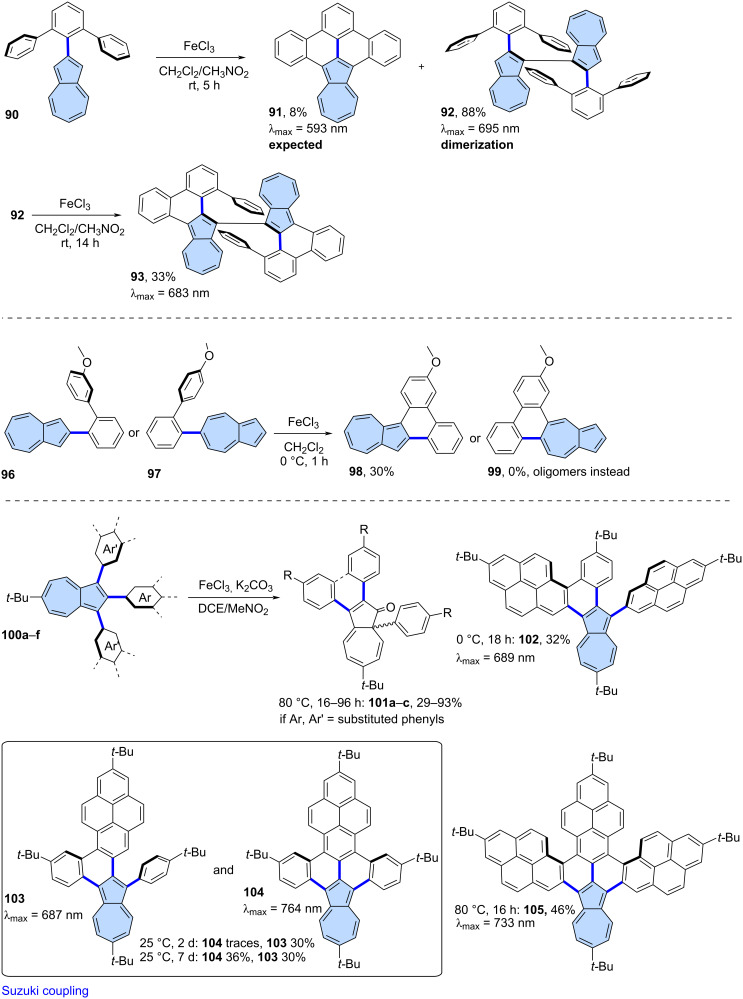
Synthesis of azulene-embedded PAHs using oxidation of substituents around the azulene core.

**Scholl-type oxidation:** The Scholl-type oxidation has also been employed also for azulene-embedded PAHs. where it was used to fuse substituents around the already existing azulene moiety in the direct precursor. However, such reactions often lead to suboptimal results in terms of yield and selectivity. Positions 1 and 3 of the azulene moiety are the most electron-rich, and pristine azulene is known to form 1,3-polyazulene upon oxidation [67], which may hinder the formation of the desired fused products. For instance, Itami and co-workers [68] reported that the oxidation of compound **92** resulted in the expected fully fused product **93**, but only in 8% yield after oxidation with FeCl_3_, while the major product was 1,1′-biazulene **94**, obtained in 88% yield (Scheme 14). Compound **94** could further be oxidized using FeCl_3_ to yield the partially fused chiral compound **95**. More recently, Morin and co-workers explored various strategies to achieve π-extended azulenes [69]. Amon other approaches, the group tested the Scholl-type oxidation of precursors **96** and **97**. However, the reaction yield was low for **96** when position 1 of azulene was involved in oxidation and fused azulene **98** was isolated only in 30% yield. The attempts to fuse position 5 in case of **97** resulted exclusively in oligomeric products and no **99** was observed.

One way to address the problem of the reactive positions (1 and 3) of the azulene unit is by blocking them in the precursor. Pigulski and co-workers explored Scholl-type oxidation of 1,2,3-triarylazulenes **100a**–**f** using FeCl_3_ as the oxidant (Scheme 14) [70]. The use of K_2_CO_3_ as an additional base was necessary because residual moisture, in the presence of FeCl_3_, led to the protonation of the starting azulenes. Interestingly, when azulenes were substituted exclusively with phenyl groups, no desired product was formed, instead surprisingly a 1,2-phenyl shift occurred, yielding azulen-1(8a*H*)-ones **101a**–**c**. However, when one or more of the substituents were replaced with a 2-pyrenyl group, partially fused (**102**, **103**) or fully fused (**104**, **105**) π-extended azulenes were obtained. This synthetic approach enabled the synthesis of a series of ‘true’ aromatic π-extended azulenes, which exhibited red-shifted azulene-like optical absorption, reaching into the NIR region.

**Scheme 15:**
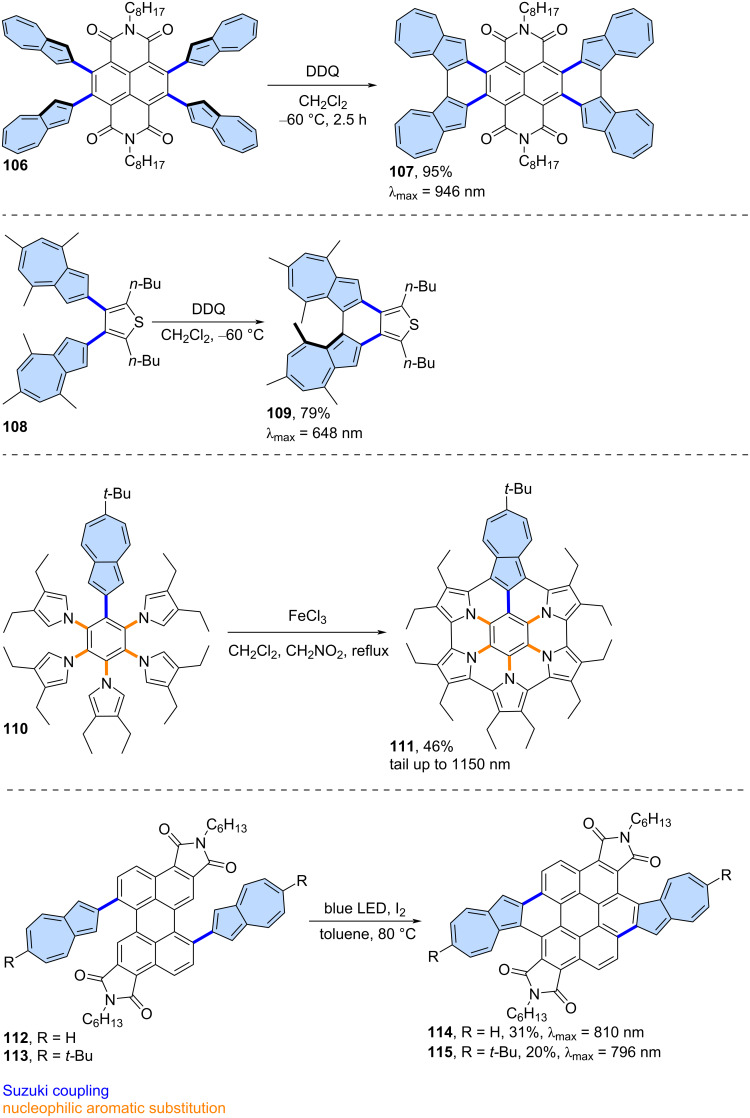
Synthesis of azulene-embedded PAHs using the oxidation of reactive positions 1 and 3 of azulene subunits.

The facile oxidation of positions 1 and 3 of the azulene moiety might be used as an advantage in the synthesis of azulene-embedded PAHs. The intramolecular oxidation of azulene units is particularly efficient when performed in an electron-deficient system, as demonstrated by Tani and co-workers [71] in their synthesis of azulene-fused tetracene diimide **107** from precursor **106** (Scheme 15). Oxidation with DDQ gave the target product in very high yield (95%). Interestingly, compound **107** contains four azulene subunits and exhibits strongly red-shifted azulene like optical absorption, with a maximum at 946 nm. A similar approach was demonstrated by the same group in the synthesis of azulene-based helicene **109**, achieved by oxidizing precursor **108** using DDQ [72]. Notably, PAH **109** forms an air-stable radical cation after oxidation. A similar intramolecular oxidation of two adjacent azulene units was also reported with the use of FeCl_3_ as an oxidant [73] or in one step during Suzuki coupling between 1,8-dibromonaphthalene and borylated azulene [74].

The ease of oxidation at positions 1 and 3 of azulene was utilized by Uno and co-workers in the synthesis of azulene-fused azacoronene **111** [75]. Oxidation of **110** using FeCl_3_ gave the nanographene **111** in 46% yield. Compound **111** exhibits red-shifted azulene-like NIR absorption with tail up to 1150 nm in CS_2_ and contains an aromatic azulene subunit. A similar oxidation can also be carried out under photochemical conditions, as demonstrated by Zhang and co-workers [76]. Precursors **112** and **113** were oxidized using I_2_ under blue LED irradiation, yielding bisimides **114** and **115** in 31% and 20% yield, respectively. Both PAHs **114** and **115** exhibit NIR optical absorption, with azulene subunits that retain their aromatic properties.

**Scheme 16:**
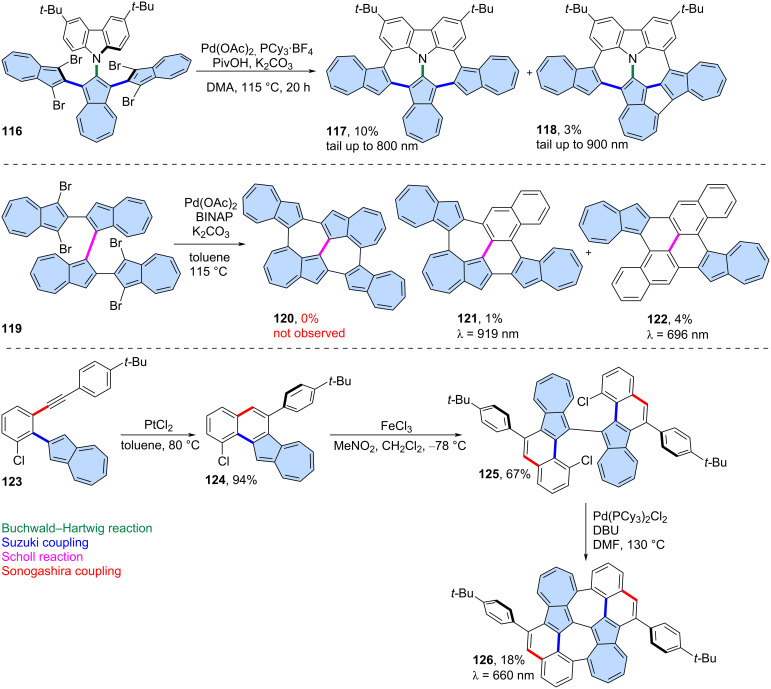
Synthesis of azulene-embedded PAHs using intramolecular C–H arylation.

**Intramolecular C–H arylation:** The intramolecular, palladium-catalysed C–H arylation can also serve as an effective tool for the fusion of azulene-embedded nanographenes. Liu and co-workers reported the synthesis of azulene-embedded nanographenes **117** and **118** using this method (Scheme 16) [77]. Precursor **116** was designed to undergo a four-fold intramolecular C–H arylation, but due to dehalogenation, only the products of double (**117**) and triple C–H arylation were isolated in 10% and 3% yields, respectively. The optical absorption of **117** and **118** reaches the desired NIR region, owing to the retention of the azulene-like electronic structure within the azulene subunits. The same group applied this strategy to precursor **119**, however, the target PAH **120** was not observed after the reaction (Scheme 16) [78]. Instead, products of a skeletal arrangement of one azulene moiety **121** and two azulene moieties **122** were isolated in low yields (1% and 4%, respectively). Plausible mechanisms of such a cyclopenta[*ef*]heptalene to phenanthrene rearrangement were proposed by the authors and involve the arenium ion pathway or Pd catalyst pathway. Both **121** and **122** exhibit typical azulene-like red-shifted absorption due to almost forbidden S_0_→S_1_ transition.

Liu and co-workers reported also an isomer of bischrysene containing two azulene subunits (Scheme 16) [79]. Precursor **125** was obtained through PtCl_2_-catalysed intramolecular annulation of alkyne **123**, followed by a Scholl-type oxidation of **124**. Finally, double intramolecular C–H arylation catalysed by Pd(PCy_3_)_2_Cl_2_ gave non-alternant PAH **126** in an 18% yield. Interestingly, according to the calculated NICS values, all heptagons of **126** lost their aromatic character. Later, it was reported that PAH **126** exhibits *anti*-Kasha fluorescence [80] from the S_3_ state in the range of 410–470 nm upon excitation at 370 nm. This was well verified by femtosecond time-resolved absorption spectroscopy (fs-TAS), with corresponding high-energy excited state absorption bands observed at 660 nm.

**Scheme 17:**
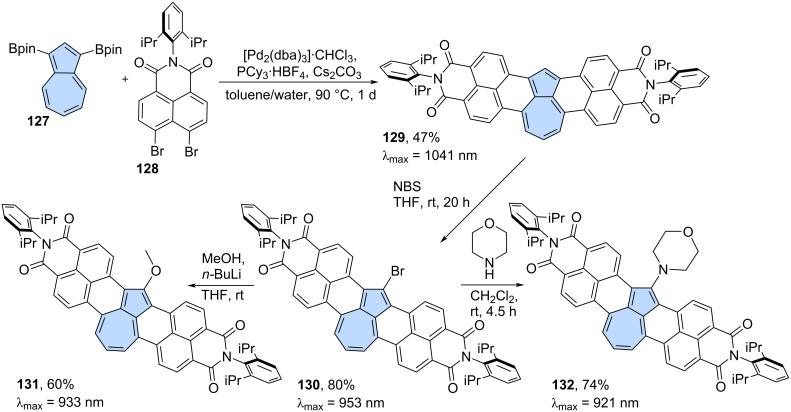
Synthesis of an azulene-embedded isomer of terylenebisimide using tandem Suzuki coupling and C–H arylation.

Würthner and co-workers developed a cascade [3 + 3] annulation strategy, where Suzuki cross-coupling is followed by C–H arylation, and applied it to various electron-deficient nanographenes [81–84]. This strategy can also be applied to non-alternant PAHs. For example, azulene **127** reacts effectively with imide to yield the non-alternant PAH **129** in 47% yield (Scheme 17) [85]. The resulting non-alternant isomer of perylenebisimide **129** exhibits strongly red-shifted absorption (λ_max_ = 1041 nm) and an azulene-like electronic structure. The optical absorption of PAH **129** is strongly bathochromically shifted compared to isomeric terrylenebisimide (λ_max_ = 650 nm) [86] and even larger rylene bisimides like hexarylenebisimide (λ_max_ = 953 nm) [87]. Bisimide **129** might be regioselectivily brominated using NBS, yielding PAH **130** in 80%. The bromide **130** undergoes nucleophilic substitution with methoxide or morpholine, giving the corresponding substitution products **131** and **132** in 60% and 74%, respectively. Very recently, during revision of this work, Aratani and co-workers reported the use of this strategy in the synthesis of two azulene-embedded isomers of perylene monoimide [88].

**Scheme 18:**
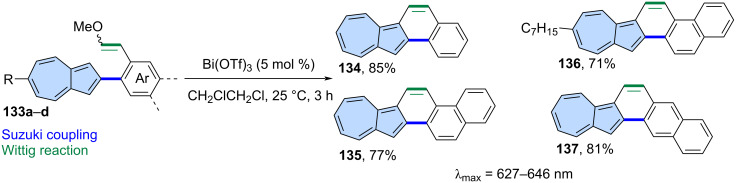
Synthesis of azulene embedded PAHs using a bismuth-catalyzed cyclization of alkenes.

**Cyclization of alkenes:** A bismuth-catalysed cyclization of alkenes has been demonstrated as an efficient synthetic tool for the creation of benzenoid PAHs [89]. Murai and co-workers applied this approach to the synthesis of azulene-embedded nanographenes (Scheme 18) [90]. Vinyl ethers **133a**–**d** were cyclized using Bi(OTf)_3_ in 1,2-dichloroethane giving PAHs **134**–**137** in high yields (71–85%). All the new PAHs exhibit characteristics typical for “true” π-extended azulenes, such as azulene-like optical absorption and narrow HOMO–LUMO gaps. In addition, compounds **134**–**137** show reversible stimuli-responsiveness against the acid–base reaction.

**Cyclization of alkynes:** The extension of π-conjugation in polycyclic aromatic hydrocarbons (PAHs) through alkyne-benzannulation reactions has become an increasingly popular method in recent years [91]. Such benzannulations can be mediated by Brønsted acids, Lewis acids or transition metals, and have been applied to a wide range of PAHs [92] and graphene nanoribbons [93]. More recently, this synthetic strategy has been independently employed by several research groups for the synthesis of non-alternant azulene-embedded PAHs. Typically, modular synthesis of direct precursors can be achieved using Suzuki and Sonogashira cross-coupling reactions.

**Scheme 19:**
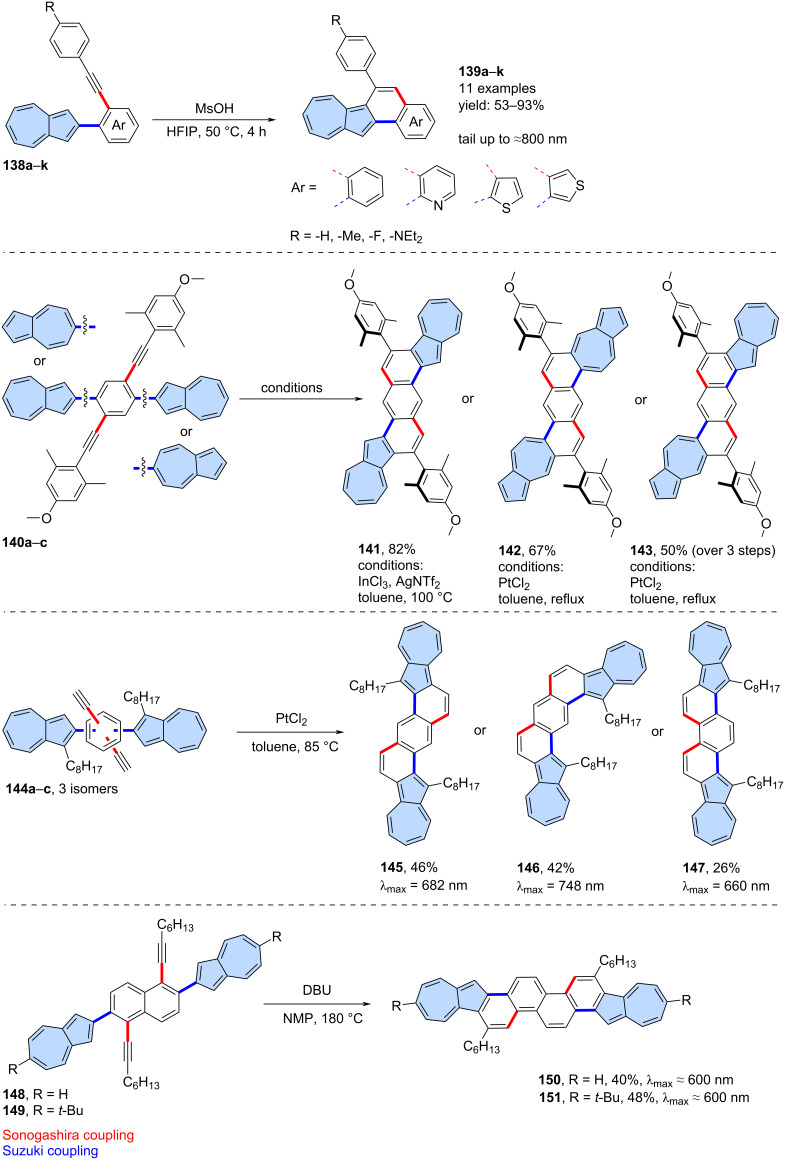
Synthesis of azulene-embedded nanographenes using intramolecular cyclization of alkynes.

One of the first examples was the synthesis of diazuleno[1,2,3-*cd*:10,20,30-*fg*]pyrene, which was later subjected to on-surface transformations [94]. More recently, a more general approach was reported by Langer and co-workers, who described the simple single benzannulation of a series of precursors **138a**–**k** (Scheme 19) [95]. The reaction was mediated by MsOH and carried out in hexafluoroisopropanol (HFIP), yielding the final products (**139a**–**k**) in 53–93% yield. In general, the absorption spectra of the products show a typical “azulene-like” fine-structured low-energy absorption profile. Similarly, Morin and co-workers reported a similar approach to synthesize PAHs with two embedded azulene subunits (Scheme 19) [69]. Three precursors **140a**–**c** were annulated using InCl_3_/AgNTf_2_ or PtCl_2_ yielding azulene-embedded nanographenes **141**–**143**.

A similar approach was employed by Xin and co-workers in the synthesis of isomeric π-scaffolds (Scheme 19) [96]. Precursors **144a**–**c** were annulated using PtCl_2_, yielding target PAHs **145**–**147** in yields ranging from 26% to 46%. Compounds **141**–**143** and **145**–**147** can undergo a two-fold protonation process, resulting in the formation of two tropylium fragments within a single molecule. Additionally, they exhibit typical azulene-like optical absorption and the aromatic properties characteristic of azulene subunits.

Liu and co-workers extended this chemistry to substituted naphthalene derivatives which led to chrysene fused with two azulene moieties (Scheme 19) [97]. Precursors **148** and **149** were annulated using DBU (1,8-diazabicyclo(5.4.0)undec-7-ene) in NMP (*N*-methyl-2-pyrrolidone) at 180 °C. The resulting PAHs **150** and **151** were isolated in relatively good yields (40% and 48%, respectively) and exhibited typical azulene-like optical absorption. The UV–vis absorption spectra, fluorescence properties and ^1^H NMR spectroscopy, indicate that **150** and **151** can be protonated to form the corresponding tropylium cation and consecutive dication under acidic conditions, with reversible protonation−deprotonation capabilities. Additionally, new OFET-based acid vapor sensors were developed from **150** by synergistically utilizing its charge transport and protonation−deprotonation properties.

**Scheme 20:**
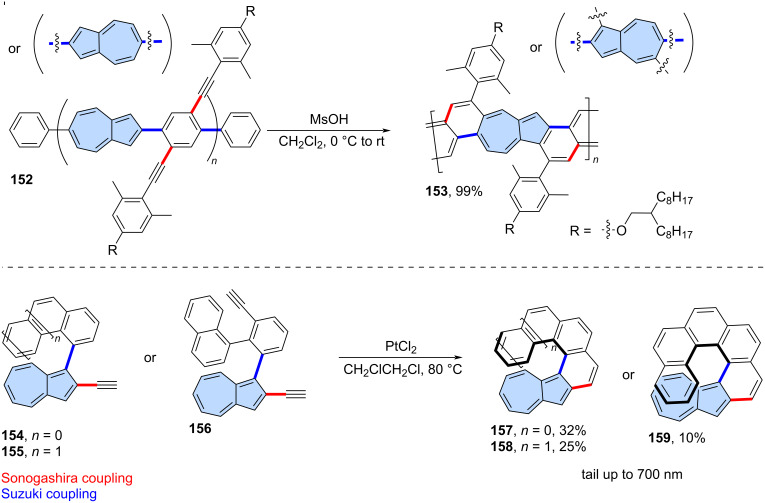
Synthesis of azulene-embedded graphene nanoribbons and azulene-embedded helicenes using annulation of alkynes.

The solution-phase synthesis of a non-benzenoid nanoribbon from an azulene-containing polymer via alkyne benzannulation was reported by Morin and co-workers (Scheme 20) [98]. The starting polymer **152** was synthesized using Suzuki cross coupling and is regiorandom, meaning the orientation of the azulene units within the main chain is not defined. Polymer **152** was annulated using MsOH (methanesulfonic acid) yielding the non-alternant graphene nanoribbon **153**. The nanoribbon is soluble in common organic solvents and exhibits conductivity values up to 1.5∙10^−3^ S∙cm^−1^ when doped by TFA in the thin film state.

The serendipitous synthesis of azulene-embedded [5]helicenes was reported by Usui, Suemune, and co-workers [99]. The unexpected formation of an azulene skeleton from a benzenoid alkyne derivative occurred when a catalytic amount of PtCl_2_ was used. A more systematic approach to [5]-, [6]-, and [7]helicenes with embedded azulene units was reported recently by Gao, Yang, and co-workers (Scheme 20) [100]. Alkyne precursors **154**–**156** were annulated using PtCl_2_, yielding a series of [*n*]helicenes (*n* = 5–7) with embedded azulene units (**157**–**159**), which were isolated in relatively low yields (10–32%). The incorporation of the azulene subunit into helicenes causes significant perturbation in the molecular electronic structure, resulting in the dark cyan or green colours of **157**–**159** and azulene-like weak absorption due to S_0_→S_1_ transition. Strong chiroptical responses were revealed by ECD spectra, with the maximum |*g*_abs_| values reaching 0.022 (at 421 nm) and 0.021 (at 427 nm) for **158**, and **159**, respectively. These values are among the highest |*g*_abs_| values of helicenes in the visible range.

**Scheme 21:**
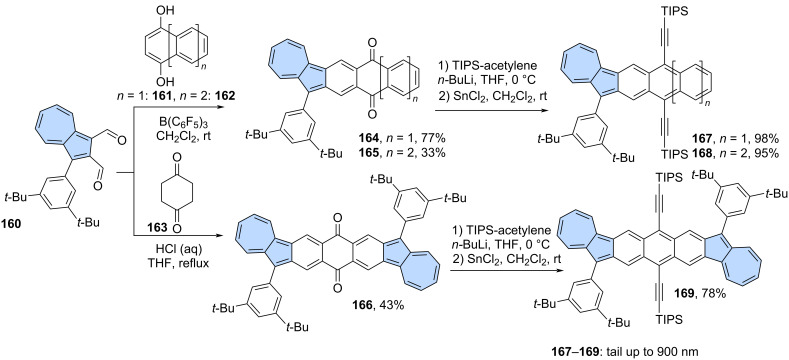
Synthesis of azulene-fused acenes.

**Miscellaneous reactions:** The scope of reactions that can be used as the final fusion step when azulene-containing precursors are employed is not limited to those described above. A synthetic strategy involving condensation followed by the reaction of the resulting 1,4-dienone with metal acetylides and dehydration is a commonly used tool for the synthesis of (hetero)acenes [101]. Jiang and co-workers applied this approach to azulene-embedded isomers of pentacene, hexacene and heptacene (Scheme 21) [102]. First, the carbon scaffolds of the target acenes were constructed by condensation of dialdehyde **160** with compounds **161**–**163** yielding diketones **164**–**166**. Next, diketones **164**–**166** were subjected to nucleophilic addition reaction by lithiated triisopropylsilyl (TIPS) acetylene, followed by SnCl_2_-mediated reduction of the intermediate diols. Finally, azulene-embedded isomers of pentacene (**167**), hexacene (**168**) and heptacene (**169**) were isolated in very good yields. Compounds **167**–**169** exhibit excellent photostability under ambient air and light conditions, as compared to their isoelectronic acene counterparts, and red-shifted azulene like optical absorption with tail up to 900 nm.

**Scheme 22:**
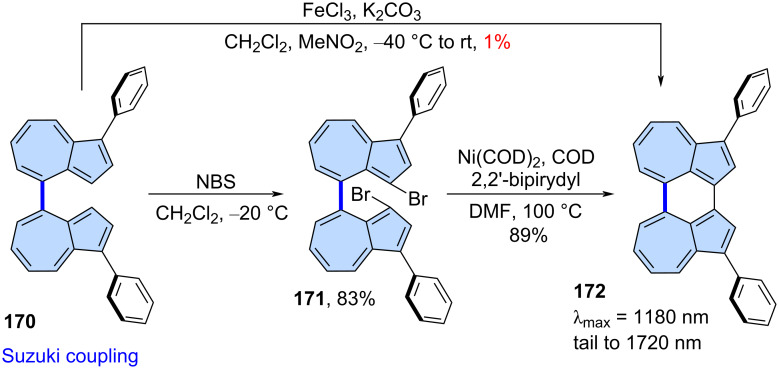
Synthesis of non-alternant isomer of perylene **172** using Yamamoto-type homocoupling.

The Yamamoto homocoupling reaction catalysed by low-valent nickel compounds [103] may be used instead of Scholl-type oxidation in the synthesis of azulene-embedded PAHs. Yamada and co-workers very recently reported the synthesis of azulene dimer **172** (Scheme 22) [104]. Initially, the authors attempted to directly oxidize **170** to **172** using Scholl reaction. However, compound **172** was isolated in only 1% yield. As an alternative, they brominated **170** to form **171**, followed by Yamamoto-type coupling using Ni(COD)_2_ and 2,2’bipirydyl (COD = 1,5-cyclooctadiene), which produced **172** in high yield (89%). Interestingly, the fusion of two azulene units at *peri*-position induces the significant orbital interaction, resulting in a very narrow HOMO–LUMO gap in **172**. Consequently, **172** exhibits NIR absorption properties (λ_max_ = 1180 nm, tail to 1720 nm) and reversible redox behaviours (electrochemical gap 1.07 eV) which is impressive for such small π-scaffold.

**Formation of carbon–heteroatom bonds:** Although the primary focus of this review is the synthesis of all-carbon azulene-embedded nanographenes, some carbon–heteroatom fusion reactions are also worth mentioning. In general, azulene-fused heteroaromatics are rare due to the limited synthetic methods available, although some N- or BN-nanographenes are known.

**Scheme 23:**
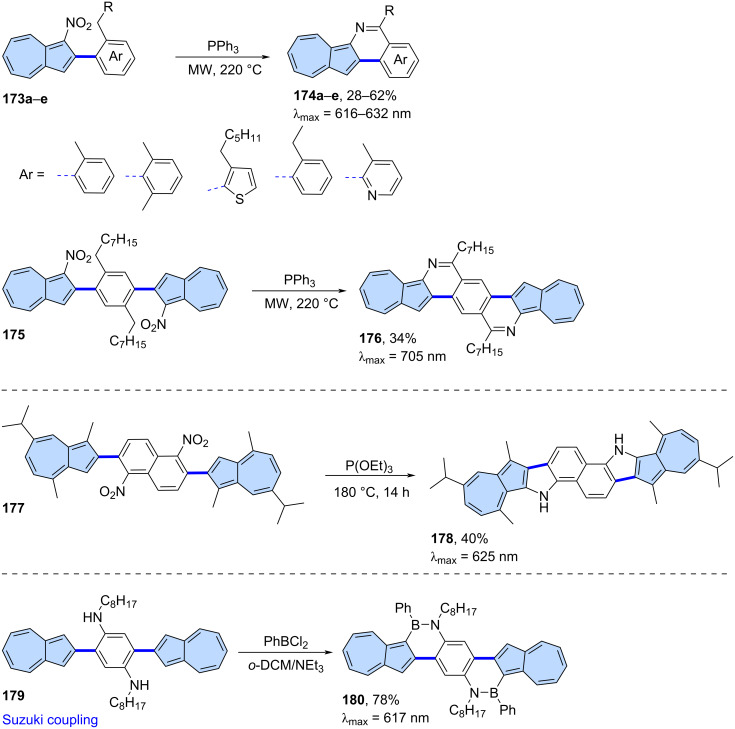
Synthesis of N- and BN-nanographenes with embedded azulene unit(s).

An eﬃcient synthesis of azulene−pyridine-fused heteroaromatics was reported by Swager and co-workers (Scheme 23) [105]. A series of monoazulene PAHs **174a**–**e** was obtained from 1-nitroazulene precursors **173a**–**e** using triphenylphosphine, instead of the expected Cadogan reaction products. This synthetic approach also works for precursors containing two azulene subunits, ultimately yielding PAH **176** in 34% yield. The results showed that these hetero-aromatics display strong aromaticity with rigid planar π-structures and exhibit weak azulene-like S_0_→S_1_ transition absorptions in the visible regions. Single-crystal ribbons of **176** exhibit p-type semiconducting behaviour with hole mobilities of up to 0.29 m^2^ V^−1^s^−1^. Typical Cadogan products can be obtained when the NO_2_ group is localized in the benzenoid part of the precursor (Scheme 23) [106]. As a result, the reaction of precursor **177** with P(OEt)_3_ gave fused π-scaffold **178** in 40% yield. Compound **178** features a highly planar geometry, narrow optical band gaps, *anti*-Kasha fluorescence, and reversible stimuli-responsiveness to acid and base. Gao and co-workers demonstrated that also BN heterocycles can be obtained using similar types of precursors (Scheme 23) [107]. Compound **179** was reacted with PhBCl_2_ to yield BN heterocycle **180** in 78% yield. Compound **180** exhibits high sensitivity for the visual detection of fluoride ions and undergoes an unexpected deboronization reaction upon the addition of TFA.

### On-surface synthesis

Recently, the field of on-surface chemistry has made significant progress, with the successful development of complex metal-catalyzed on-surface reactions that are not accessible through classical solution-based organic chemistry [108–109]. Ultra-high vacuum (UHV) conditions on metallic surface allow to observe chemical species which are very reactive and impossible to isolate using classical chemical synthesis. Therefore, it is not surprising that such synthetic techniques have been applied to the synthesis of azulene-embedded nanographenes. One of the main challenges in this area is that the outcome of reactions is often difficult to predict, and various skeletal rearrangements can occur under typical on-surface reaction conditions.

**Scheme 24:**
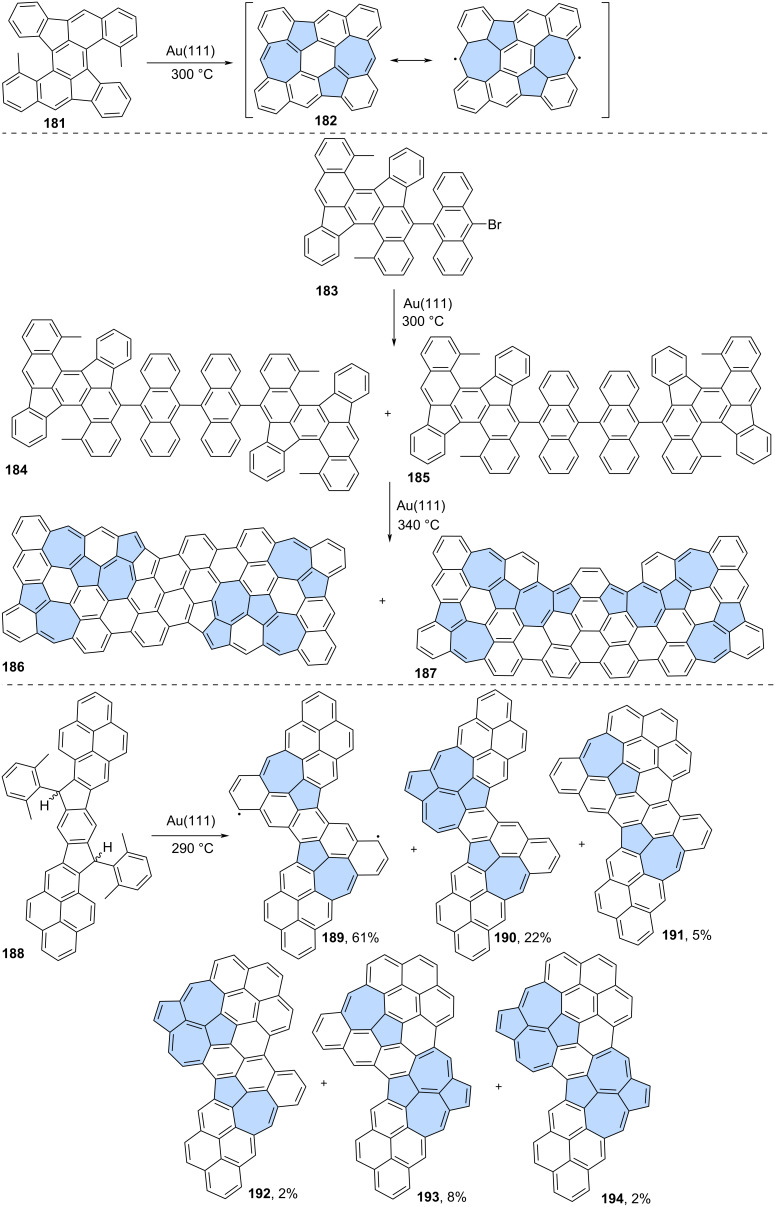
On-surface synthesis of azulene-embedded nanographenes from benzenoid precursors via dehydrogenation of methyl groups.

The synthesis of most of the reported azulene-embedded PAHs involves the generation of azulene moieties on-surface. This means that the precursors obtained through traditional solution chemistry are typically benzenoid hydrocarbons. These precursors are usually dehydrogenated on the surface, leading to the formation of formal azulene subunits. A good example of this strategy is the reaction reported by Feng and co-workers (Scheme 24) [110]. Precursor **181** was annealed on an Au(111) surface at 300 °C, resulting in PAH **182** with two embedded azulene subunits. Spin-polarized density functional theory calculations predicted that PAH **182** would exhibit an open-shell singlet ground state, as it contains five Clar sextets, compared to only two in the closed-shell structure. The same group later proposed an extension of this strategy [111]. Precursor **183**, which contains subunit **181**, was first annealed at 300 °C giving two rotamers **184** and **185** which are products of an Ullmann-type dimerization (Scheme 24). Further heating on the Au(111) surface led to products with partial skeletal rearrangement, driven by intramolecular structural strain. Both nanographenes, **186** and **187**, contain six formal azulene subunits and exhibit nearly planar geometry. However, theoretical analysis of NICS values revealed that none of the azulene subunits exhibits the characteristic azulene-like aromaticity. Additionally, both **186** and **187** show moderate open-shell biradical character, according to theoretical calculations.

A similar strategy, leading to different types of skeletal rearrangements, was reported by Ma and co-workers (Scheme 24) [112]. The reaction of precursor **188** at 290 °C on Au(111) surface produced a series of isomeric products **189**–**194**, which contains azulene and/or Stone–Wales type of defects. The main product, nanographene **189**, is formed via oxidative ring-closure of the four methyl substituents of precursor **188** after annealing. In contrast, all the other observed PAHs **189**–**194** result from oxidative ring-closure and skeletal ring-rearrangement reactions. Theoretical calculations revealed that nanographene **188** possesses an antiferromagnetic open-shell singlet ground state, whereas the other products do not.

**Scheme 25:**
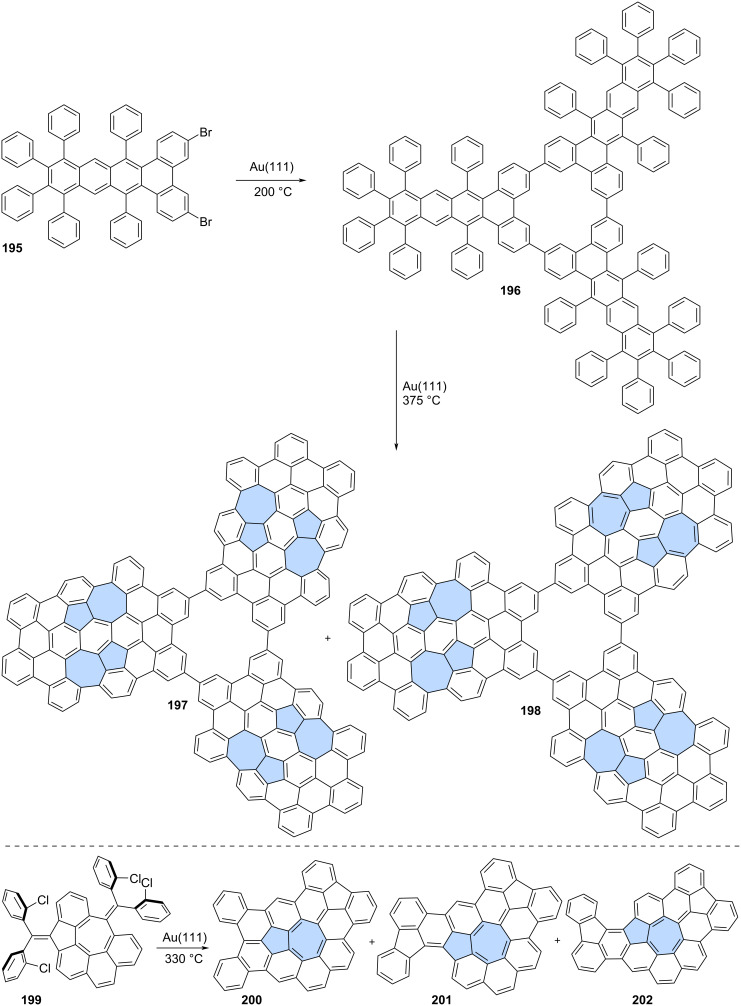
On-surface synthesis of azulene-embedded nanographenes from benzenoid precursors.

Peña and co-workers reported a two-step on-surface synthesis of impressive propeller-shaped nanographenes **196** and **197** (Scheme 25) [113]. First, benzenoid precursor **195** underwent Ullmann-type cyclotrimerization on an Au(111) surface at 200 °C, resulting in compound **196**. PAH **196** was then further heated to 375 °C, which triggered dehydrogenation and the formation of two isomeric compounds **197** and **198**. Both **197** and **198** possess six azulene subunits and an [18]annulene core. The creation of azulene moieties follows a novel cyclodehydrogenation pattern in conjoined cove regions, leading to the formation of two new C–C bonds and the relaxation of the twisted regions into a flat-lying molecule on the surface.

Ruffieux and co-workers recently reported a synthetic approach toward tetrabenzo-fused circumazulene starting from precursor **199** (Scheme 25) [114]. However, the desired circumazulene was not detected, and instead, products of some additional annulations were observed (**200**–**202**). The more planar structure of nanographenes **200**–**202** likely drives the process toward more annulated configurations. Theoretical calculations of the studied azulene-embedded PAHs indicated a strong antiaromatic character of the inner nonbenzenoid rings, particularly heptagonal rings, in contrast to pristine azulene.

**Scheme 26:**
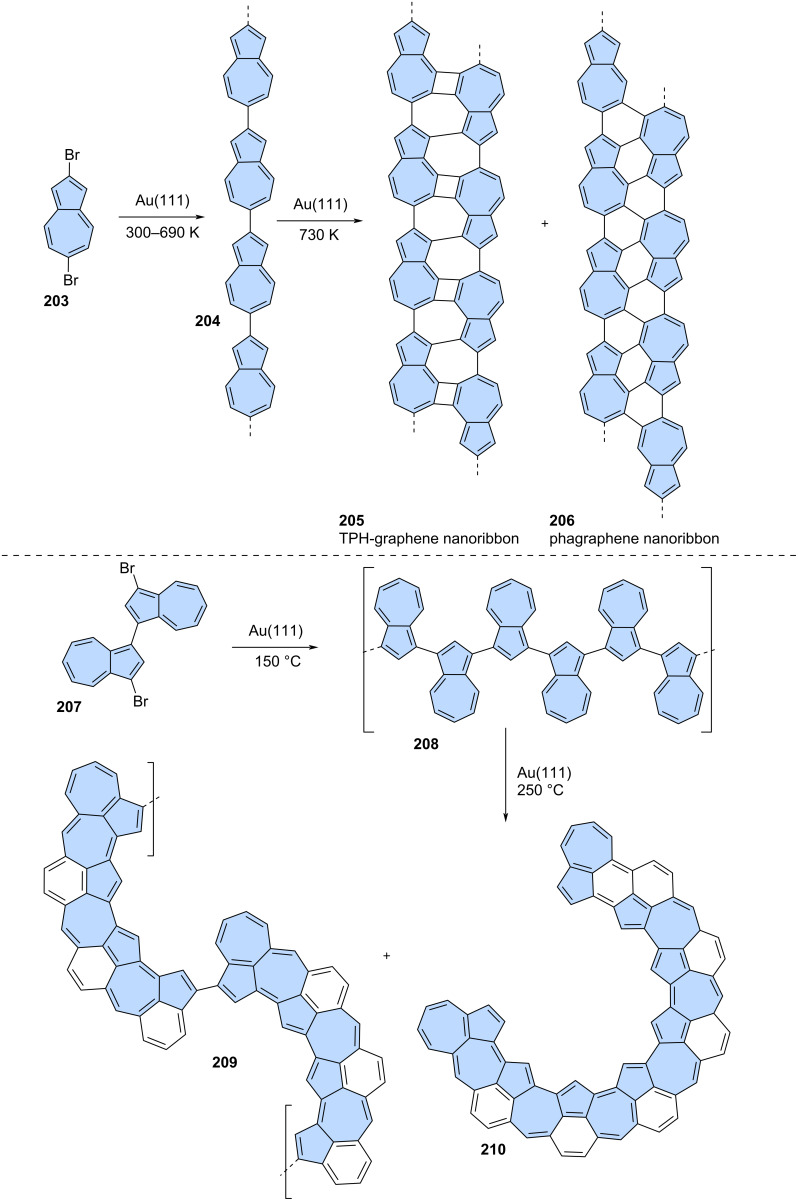
On-surface synthesis of azulene-embedded nanoribbons.

Dihalogenated precursors offer the potential to obtain polymeric structures through on-surface chemistry. A notable example was reported by Ebeling and co-workers (Scheme 26) [23]. First, simple 2,6-dibromoazulene (**203**) was annealed on an Au(111) surface, leading to the formation of 2,6-polyazulene chains **204**. Upon heating these chains to 730 K, laterally fused chains were observed. The distinctive phagraphene nanoribbon **205** and the THP-graphene nanoribbon **206** were formed. This transformation provides solid evidence that large fragments of non-alternant analogues of graphene can be synthesized from simple precursors. Similarly, Müllen and co-workers applied an analogous strategy for 3,3'-dibromo-1,1'-biazulene **207** (Scheme 26) [115]. First, biazulene **207** was polymerized to yield oligoazulene **208**. However, further annealing at 250 °C did not result in the expected products of simple fusion, but rather a mixture of various non-benzenoid PAHs due to skeletal rearrangements. Initially, polymeric structures where fused fragments were linked by single C–C bonds were observed (**209**). Additionally, fully fused PAH **210** was identified using STM. Theoretical calculations revealed that the hexagons and most of the heptagons in **210** are nonaromatic, while the pentagons, especially those in the aceheptalene subunit, are highly aromatic.

### Optical and electronic properties

Analyzing the optical and electronic properties of the diverse range of azulene-embedded nanographenes discussed in this review presents a significant challenge, as many of the original studies lack comprehensive data. In numerous cases, fluorescence characteristics were not thoroughly examined, and time-dependent density functional theory (TD-DFT) calculations – essential for accurately identifying the S_0_→S_1_ electronic transitions – were not reported. Nevertheless, certain general structure–property relationships can still be proposed for these systems. Representative examples of polycyclic aromatic hydrocarbons (PAHs) featured in this review are summarized in Table 1, which provides key data on their lowest-energy electronic transitions (S_0_→S_1_), fluorescence behavior, and first reduction/oxidation potentials. Several structural factors are particularly influential in determining properties such as near-infrared (NIR) absorption and narrow electrochemical gaps. These include: (1) the presence or absence of an azulene-like electronic structure; (2) the degree of aromaticity within the azulene subunit; and (3) the biradical character of molecule.

**Table 1:** Optical and electronic properties of selected azulene-embedded nanographenes.

Structure	λ_max_^Abs^ (ε)^a^ [nm]/[cm^−1^M^−1^]	λ_max_^Em^ [nm]	*E*_1/2_^ox^/*E*_1/2_^red b^ [V]	*E*_g_^c^ [V]	Ref.

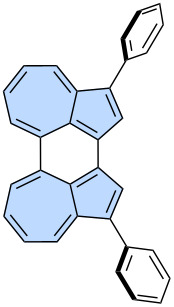 **172**	1180 (521) tail to 1720 nm (CCl_4_)	–	−0.10/−1.17 (PhCN)	1.07	[104]
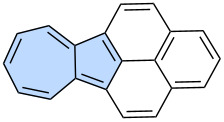 **22**	1010 (98)	–	–	–	[37]
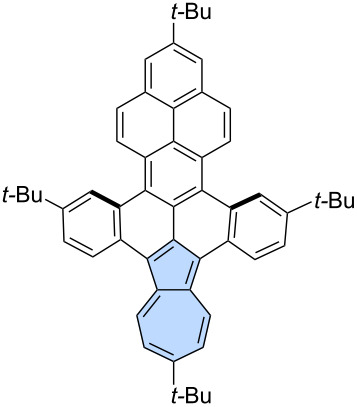 **104**	764 (450) (CH_2_Cl_2_)	–	0.10/−1.34	1.81	[70]
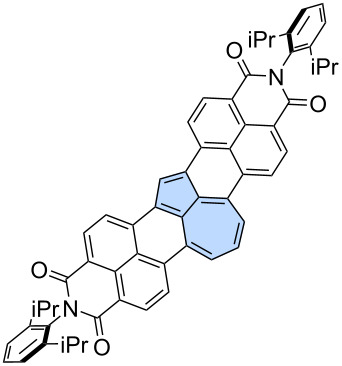 **129**	1041 (4500) (CH_2_Cl_2_)	–	0.59/−1.03 (CH_2_Cl_2_)	1.62	[85]
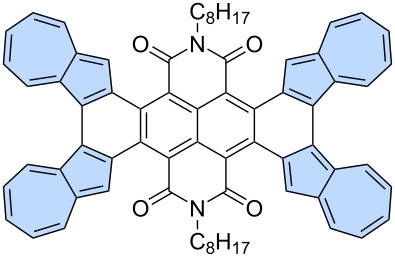 **107**	946 (2500) (THF)	–	0.19/−0.77 (PhCN)	0.96	[71]
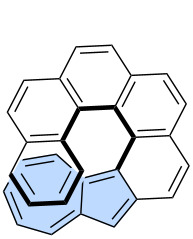 **159**	tail up to 700 nm (CH_2_Cl_2_)	463^d^ (Φ_FL_ < 0.1%) (CH_2_Cl_2_)	0.09/−1.84 (onsets, CH_2_Cl_2_)	1.93	[100]
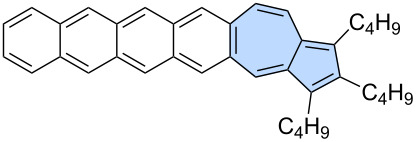 **60**	680 (weak) (CH_2_Cl_2_)	529^d^ (CH_2_Cl_2_)	0.06/−1.69 (CH_2_Cl_2_)	1.75	[55]
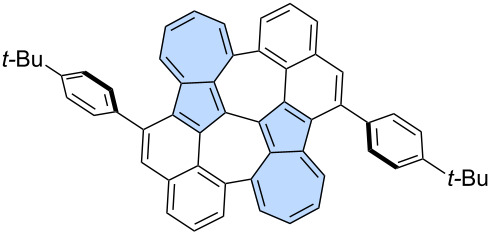 **126**	≈660 (weak) (THF)	410^d^ (THF)	0.12/−1.45 (THF)	1.55	[79–80]
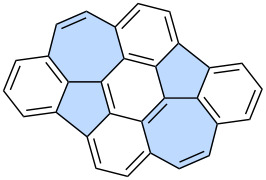 **29**	666 (15800) (THF)	400^d^, 670 (THF)	0.22/−1.74 (*o*-DCB/CH_2_Cl_2_)	1.96	[42]
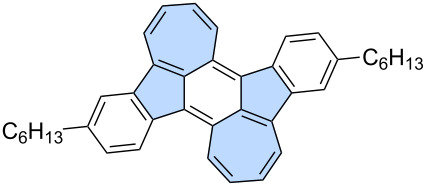 **24**	997 (weak) (CH_2_Cl_2_)	–	−0.11/−1.34 (CH_2_Cl_2_)	1.45	[39]
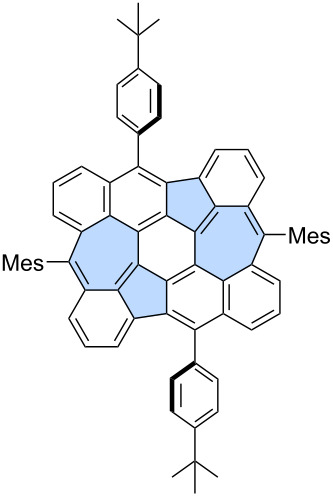 **26**	936 (weak) (CH_2_Cl_2_)	–	0.11/– (CH_2_Cl_2_)	–	[40]
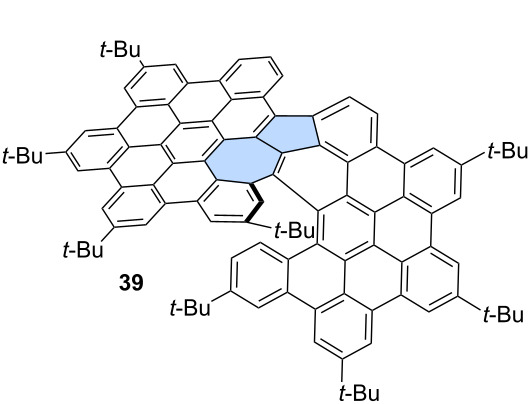	629 (CH_2_Cl_2_)	–	0.31/−1.69 (CH_2_Cl_2_)	2.00	[47]
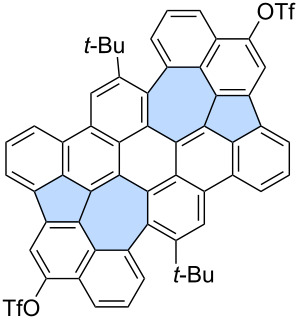 **42**	628 (CH_2_Cl_2_)	648 (Φ_FL_ = 20%) (CH_2_Cl_2_)	0.42/−1.67 (CH_2_Cl_2_)	2.09	[48]

^a^λ_max_ – maximum of the lowest energy electron transition, ε – extinction coefficient; ^b^First oxidation and reduction potentials versus Fc/Fc^+^ couple; *^c^E*_g_ = *E_1/2_*^ox^ – *E_1/2_*^red^; ^d^*anti*-Kasha emission.

Purely hydrocarbon PAHs containing ‘true’ aromatic azulene subunits (e.g., compounds **172**, **22**, and **104**) can exhibit remarkably red-shifted absorption, even when incorporated into relatively small π-conjugated frameworks. Through careful molecular design – retaining the azulene-like electronic structure and promoting spatial separation of the HOMO and LUMO orbitals – it is possible to achieve exceptionally narrow optical gaps. A striking example is the perylene isomer **172**, which displays absorption extending to 1720 nm and an electrochemical gap of just 1.07 V [104]. The presence of two parallel azulene moieties in this structure results in an enhanced dipole moment (1.97 D) compared to pristine azulene (1.28 D). This parallel alignment appears to be critical for achieving a narrow optical gap: in contrast, a recently reported azulene dimer with antiparallel azulene units exhibits a lowest-energy transition at 680 nm [116], comparable to that of pristine azulene. However, the molar extinction coefficients (ε) of compounds **172**, **22**, and **104** are relatively low (below 1000 M^−1^ cm^−1^), reflecting the partially forbidden nature of the S_0_→S_1_ transitions. Incorporating strongly electron-withdrawing imide groups can enhance the intensity of these transitions, as demonstrated in the cases of the terylene bisimide isomer **129** (λ_max_ = 1041 nm, ε = 4500 M^−1^ cm^−1^) [85] and bisimide **107** (λ_max_ = 946 nm, ε = 2500 M^−1^ cm^−1^) [71]. Notably, none of the aforementioned compounds exhibit Kasha-type or *anti*-Kasha fluorescence.

Some azulene-embedded PAHs exhibit *anti*-Kasha fluorescence, akin to that observed in pristine azulene [117]. Notable examples include two series of compounds that feature aromatic azulene subunits: isomers of [*n*]helicenes (*n* = 5, 6, 7) [100] and [*n*]acenes (*n* = 2–6) [55], all of which display *anti*-Kasha fluorescence. Selected representatives from both series are listed in Table 1; for instance, [7]helicene **159** emits at 463 nm, while [6]acene **60** emits at 529 nm. Azulene-embedded nanographenes containing only ‘formal’ (structurally defined but not truly aromatic) azulene subunits can also exhibit *anti*-Kasha emission. PAH **126** shows *anti*-Kasha emission at 410 nm, whereas compound **29** displays dual emission behavior – both *anti*-Kasha (400 nm) and Kasha-type (670 nm) fluorescence. Nanographenes with formal azulene subunits and significant biradical character (e.g., compounds **24** and **26**) typically show strongly red-shifted optical absorption but no fluorescence.

Interestingly, when the benzenoid framework dominates and the formal azulene unit acts primarily as a structural linker, even large nanographenes may not exhibit red-shifted absorption. PAHs **39** and **42** exemplify this behavior: despite their extended π-conjugation, both show optical absorption and electrochemical gaps characteristic of benzenoid PAHs, with either no fluorescence (compound **39**) or weak Kasha-type fluorescence (compound **42**). Lack or weak fluorescence is a typical behavior of warped, distorted benzenoid PAHs due to the fact that they can suffer from enhanced intersystem crossing [118].

## Conclusion

All modern synthetic approaches to azulene-embedded nanographenes have been summarized. These molecules demonstrate a diverse range of electronic properties depending on their specific π-conjugated scaffold. While some PAHs contain aromatic „true” azulene subunits, while the others exhibit biradical properties or benzenoid part of molecules has dominant impact on the properties. Moreover, the incorporation of azulene units into PAHs results in unique and exciting properties, including biradical character, near-infrared (NIR) absorption, stimuli responsiveness, and *anti*-Kasha emission. These characteristics make azulene-embedded nanographenes promising candidates for applications in organic electronics, optoelectronics, and molecular materials.

Despite recent progress, several challenges remain to be addressed. The number of modular synthetic strategies for azulene-embedded nanographenes is still limited, necessitating the development of more efficient and scalable approaches. Additionally, the role of serendipity, particularly in reactions such as the Scholl oxidation, continues to hinder precise control over molecular structures. A deeper mechanistic understanding of these transformations is essential for achieving predictable and reproducible outcomes. Finally, the design and synthesis of substructures related to hypothetical non-benzenoid carbon allotropes represent an exciting avenue for future research, potentially leading to the discovery of novel materials with unprecedented electronic and structural properties.

## Data Availability

Data sharing is not applicable as no new data was generated or analyzed in this study.
